# Insecticidal Activity and Free Radical Scavenging Properties of Isolated Phytoconstituents from the Saudi Plant *Nuxia oppositifolia* (Hochst.)

**DOI:** 10.3390/molecules26040914

**Published:** 2021-02-09

**Authors:** Shaza M. Al-Massarani, Ali A. El-Gamal, Adnan J. Al-Rehaily, Ebtesam S. Al-Sheddi, Mai M. Al-Oqail, Nida N. Farshori, Alden S. Estep, Nurhayat Tabanca, James J. Becnel

**Affiliations:** 1 Department of Pharmacognosy, College of Pharmacy, King Saud University, P.O. Box 2457, Riyadh 11451, Saudi Arabia; aelgamal00@yahoo.com (A.A.E.-G.); ajalreha@ksu.edu.sa (A.J.A.-R.); ealsheddi@ksu.edu.sa (E.S.A.-S.); maloqail@ksu.edu.sa (M.M.A.-O.); nidachem@gmail.com (N.N.F.); 2 Department of Pharmacognosy, Faculty of Pharmacy, Mansoura University, El-Mansoura 35516, Egypt; 3 USDA, ARS, Center for Medical, Agricultural, and Veterinary Entomology, Gainesville, FL 32608, USA; alden.estep@usda.gov (A.S.E.); james.becnel@usda.gov (J.J.B.); 4 USDA-ARS, Subtropical Horticulture Research Station, 13601 Old Cutler Rd., Miami, FL 33158, USA; nurhayat.tabanca@usda.gov

**Keywords:** *Nuxia oppositifolia*, flavonoids, phenylethanoid, triterpenes, mosquito control, biopesticides, *Aedes aegypti*, free radical scavenging, DPPH, ABTS

## Abstract

Chromatographic purification of the alcoholic extract from the aerial parts of the Saudi plant *Nuxia oppositifolia* (Hochst.), Benth., resulted in five isolated phenolic compounds. Two flavones, hispidulin (**1**) and jaceosidin (**2**), and the phenylethanoid glycosides, verbascoside (**3**), isoverbascoside (**4**), and conandroside (**5**), were identified and their chemical structures were determined by spectroscopic analyses. The insecticidal activity of compounds **1** and **2,** in addition to 11 compounds isolated in a previous research (**6**–**16**), was evaluated against the Yellow Fever mosquito, *Aedes aegypti*. Four compounds displayed adulticidal activity with LD_50_ values of 2–2.3 μg/mosquito. Free radical scavenging properties of the plant extracts and compounds (**1**–**5**) were evaluated by measuring the 1,1-diphenyl-2-picrylhydrazyl radical (DPPH) and 2,2′-azino-bis (3-ethylbenzothiazoline-6-sulfonate radical cation (ABTS^•+^) scavenging activity. All compounds exhibited notable activity, compared with the positive control, l-Ascorbic acid. This study suggests that *N. oppositifolia* could be a promising source of secondary metabolites, some with lethal adulticidal effect against *Ae. aegypti*.

## 1. Introduction

The bite of an infected *Aedes aegypti* L. mosquito can spread several dangerous diseases, such as Dengue, Zika, and Chikungunya fever. These diseases are worldwide public health problems, with particularly severe consequences in developing countries. Controlling the mosquito vector is the best method for disease prevention, and the need for new mosquitocidal agents due to resistance and regulatory loss is a serious issue [1].

Plants and their derivatives have been used for millennia in insect control because of beneficial toxic activity or repellent properties. Phytochemicals responsible for such activity are primarily secondary metabolites synthesized by plants for defense against herbivorous insects [2]. A few phytochemical and biological studies have examined the *Nuxia* (family: Buddlejaceae) for beneficial compounds and found possible candidate molecules. Secondary metabolites were isolated from the leaves and aerial parts of *N. oppositifolia*, *N. floribunda*, and *N. sphaerocephala*. [3,4,5,6]. Anti-diabetic activity of *N. floribunda* [7], and anti-inflammatory, antimalarial, and cytotoxic activity of *N. verticillata* were also previously evaluated [8,9].

*Nuxia* is represented in Saudi Arabia by two species, *N. oppositifolia* Benth and *N. congesta* Fresen. Recently, we isolated three new labdane-type diterpene acids and 11 triterpenes, including a triterpene derivative, and the common phytosterols, β-sitosterol and stigmasterol from the *n*-hexane and dichloromethane extracts of *N. oppositifolia*. The compounds demonstrated high in vitro cytotoxic activities against human cancer (cervical, lung, breast) cell lines [5]. Two of the isolated compounds, 3-oxolupenal and katononic acid, showed significant affinity at the binding sites of α-amylase and α-glucosidase and could be useful for treatment of diabetes mellitus type II [10].

In the present study, we continued our search for biologically active secondary metabolites from the native Saudi species, *N. oppositifolia*, and describe the isolation and identification of two flavones (**1** and **2**) and three phenylethanoid glycosides (**3**–**5**). Compounds **1** and **2,** as well as 11 compounds isolated in our previous research (**6**–**16**), were tested for adulticidal activity against the mosquito *Aedes aegypti*. In addition, the free radical scavenging activities of compounds (**1**–**5**) were assessed by DPPH and ABTS scavenging assays.

## 2. Results and Discussion

### 2.1. Identification of Isolated Compounds

Dichloromethane and *n*-butanol soluble extracts from the aerial parts of *N. oppositifolia* were fractionated into constituents utilizing a combination of different chromatographic techniques. This resulted in the isolation of five phenolic compounds. Spectroscopic data (1D, 2DNMR, and MS) of the isolated compounds were compared with data in the literature to identification (Figures S1–S24). We determined that the flavonoids hispidulin, CID 5281628 (**1**) and jaceosidin, CID 5379096 (**2**) [11,12], and the phenylethanoid glycosides verbascoside, CID 5281800 (β-(3′,4′-dihydroxyphenyl)ethyl-*O*-α-l-rhamnopyranosyl (1→3)-β-d-(4-*O*-caffeoyl)-glucopyranoside) (**3**), isoverbascoside, (CID 13889686, (**4**) [13,14] and conandroside, SID 24724290 (**5**) [15] were present (Figure 1). Only verbacoside has been previously reported from the genus *Nuxia* [4].

### 2.2. Adulticidal Activity

**Figure 2:**
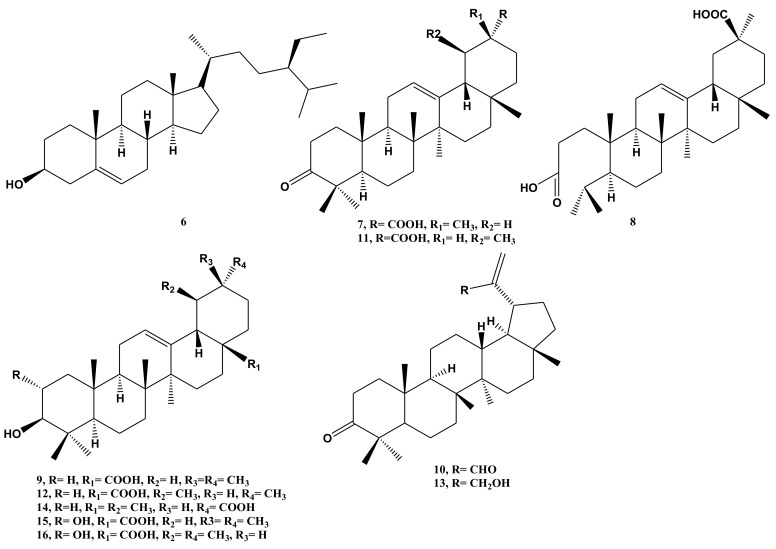
Chemical structures of the previously isolated compounds (**6**–**16**) from *N. oppositifolia*.

Thirteen compounds, including two flavonoids (**1** and **2**) isolated in the present study and eleven terpenoids identified in previous work (Figure 2), were screened at a dose of 5 μg/mosquito for 24 h mortality against female *Ae. aegypti* (Table 1). Permethrin control mortality was 37 ± 6% and 100% and at doses of 0.15 and 2.37 ng/mosquito, respectively. Solvent control (acetone) and untreated controls showed 0% mortality. Compounds **6**–**9**, isolated from *N. oppositifolia*, showed over 85% mortality at doses of 5 μg/mosquito. These compounds were further tested to calculate LD_50,_ which ranged from 2.08 to 2.22 μg/mosquito (Table 1).

The phytosterol, β-sitostosterol, has demonstrated larvicidal activity (LC_50_ of 11.49 ppm) against *Ae. aegypti* [16]. In a recent study, conducted by Tabanca et al., β-sitosterol (**6**) showed an LC_50_ value of 1.7 (1.3–2.3) ppm and LC_90_ value of 5.1 (3.4–13.8) ppm at 24 h post-treatment [17]. Glycosidation of β-sitostosterol did not reduce its mosquitocidal activity. β-sitosterol-3-*O*-β-d-glucoside exhibited 100% mortality against adult *Ae. aegypti* at a concentration of 1.25 μg/mg [18]. Moreover, oleanolic acid (**9**) caused in vitro oxidative stress and apoptosis in *Setaria digitate,* the parasite that causes filariasis [19]. To the best of our knowledge, this study is the first to report the adulticidal activities of the triterpenes (compounds **7**–**16**), as well as the flavonoids **1** and **2** against *Ae. aegypti*.

### 2.3. Free Radical Scavenging Activity

Phenolic compounds, including the flavonoids, are bioactive secondary metabolites known for a wide range of health benefits [13,20]. They have considerable antioxidant activity, as demonstrated in several in vitro and in vivo assay systems [13,21,22]. Multiple electron-donating phenolic hydroxyl groups in the flavonoid and phenylethanoid glycosides and resonance stabilization of the radicals explain this high radical scavenging activity [23,24]. The vast literature confirming antioxidant activity of phenolic compounds encouraged us to test the radical scavenging potential of *N. oppositifolia* isolates, as shown in Table 2. All samples displayed the capacity to reduce the DPPH and (ABTS^•+^) radicals with a dose-dependent inhibition. At 1000 µg/mL concentration, the activity of tested samples ranged from 75.7 ± 0.4 to 79.8 ± 1.2% and from 73.7 ± 0.3 to 77.1 ± 0.1% in the DPPH and ABTS assays, respectively. At the same concentration, the l-Ascorbic acid positive control had a scavenging activity of 90.7 ± 1.4 and 88.7 ± 2.1% in the DPPH and ABTS assays, respectively. Jaceosidin (**2**) had the lowest IC_50_ (28.0 ± 0.8 μg/mL) in the ABTS assay, compared to ascorbic acid displaying IC_50_ of 6.0 ± 0.85 μg/mL. Conandroside (**5**), with a structure similar to verbascoside but with a xylose instead of rhamnose moiety attached to the glucose, had the highest activity with a significantly (*p* < 0.005) lower IC_50_ of 27.3 ± 1.1 μg/mL than ascorbic acid (IC_50_ of 5.0 ± 0.15 μg/mL). Conandroside has limited abundance in nature and has been isolated from the families, Gesneriaceae, Lamiaceae, and Polypremaceae [25]. To the best of our knowledge, this study is the first report of the antioxidant potential of conandroside.

## 3. Materials and Methods

### 3.1. Apparatus and Chemicals

Mass determination used a Jeol JMS-700 high-resolution mass spectrophotometer, with electron impact mode ionization at 70 ev. IR spectra were recorded on JASCO 320-A spectrometer. The ^1^H- and ^13^C-NMR spectra were recorded on an Ultra Shield Plus 500 MHz (Bruker, Billerica, MA, USA) spectrometer with a TMS internal standard.

Isolation of compounds was partially accomplished by open column chromatography using silica gel, particle size 0.04–0.063 mm, Sephadex LH-20 (Fluka, Buchs, Switzerland) and porous-polymer Diaion HP-20 polystyrene resin (Mitsubishi Chemical, Tokyo, Japan). Centrifugal preparative thin layer chromatography (CPTLC) was used with a Chromatotron device (Harrison Research, Palo Alto, CA, USA). Chromatographic analysis was performed with precoated F_254_ normal and RP-18 thin-layer chromatography plates (Merck, Darmstadt, Germany), with detection at 254 or 366 nm, and by spraying with ceric sulphate reagent.

2,2-Diphenyl-1-picrylhydrazyl (DPPH) radical and ascorbic acid was acquired from Sigma-Aldrich. Reagents, chemicals, and solvents were of analytical grade, purchased from Sigma-Aldrich (St. Louis, MO, USA), Loba Chemie Pvt. Ltd. (Mumbai, India), and SD Fine Chem. Ltd. (Mumbai, India). Adult *Aedes aegypti* (Orlando1952 strain) were acquired from laboratory colonies maintained at the USDA-ARS, CMAVE, Gainesville, FL, USA.

### 3.2. Plant Material

The aerial parts of *N. oppositifolia* were collected in March 2015, in Wadi Lajab Southern in Saudi Arabia, latitude: 17°36′10.8″ N and longitude: 42°56′00.6″ E and identified at the Pharmacognosy Department, College of Pharmacy, King Saud University. A voucher specimen (# 15501) was deposited at the Pharmacognosy Department, College of Pharmacy, King Saud University for future reference.

### 3.3. Extraction and Isolation

The remaining dichloromethane fraction from our previous study (8 g) [5], was applied to a silica gel CC and eluted with increasing amounts of MeOH/CHCl_3_ to yield four major fractions (I–IV). Part of fraction II, eluted with 5% MeOH/CHCl_3_, was purified by chromatotron with 15% in *n*-hexane:acetone:acetic acid (85:15:1) to afford compound **1** in pure form (46 mg). Another portion of fraction II was purified on a Sephadex LH-20 column using MeOH/H_2_O (90:10) to afford compound **2** (45 mg). The *n*-butanol extract (70 g) was fractionated by passage through a porous-polymer Diaion HP-20 polystyrene resin column, eluted with water, then with an increasing concentration of MeOH, finally finishing with pure MeOH to afford 7 sub-fractions. Fractions 5 and 6, eluted with 60 and 80% MeOH/H_2_O, respectively, were combined after monitoring on Kiesel gel 60 F254 TLC. Further, chromatotron purification with 15% MeOH/CHCl_3_ followed by RP-18 CC (MeOH/H_2_O, 1:1) yielded compounds **3** (9 mg), **4** (11 mg), and **5** (8 mg), in pure form.

### 3.4. Adulticidal Activity

Screening and toxicities of isolated compounds were evaluated in assays using cohorts of 3–5 day old adult *Ae. aegypti* females, following previously described procedures from a large natural product screening program [26]. Briefly, compounds were dissolved to a concentration of 100 μg/μL in DMSO with vigorous vortexing or gentle heating if needed. A 10 μg/μL solution for screening was created by diluting the 100 μg/μL stock in acetone. Orlando 1952 strain mosquitoes were cold anesthetized and sorted into groups of 10 females per TK35 cup (Solo Co, USA) and maintained at 4 °C until dosing. Initial screening of compounds at 5 μg/mosq was conducted by application of a 0.5 μL droplet to the ventral aspect of each mosquito using a repeating pipettor and a 25-μL Series 7100 gas tight syringe with a blunt tip (Hamilton Syringe Co.) to avoid damaging the mosquito. Cohorts of 10 mosquitoes were dosed with a specific compound in each assay. After dosing, cups were covered with a screen mesh and secured with a rubber band. Cotton balls saturated with 10% sucrose were provided for each cup and then cups were maintained at 22 °C in an insectary. Mortality was recorded 24 h after application. Permethrin and acetone were used as positive and negative controls, respectively. Initial screening assays were conducted three times. Subsequent dose response assays (three separate assays) were conducted for the four samples that produced screening mortality above 80% in initial screening. Dose response assays used the initial 100 μg/μL stock of each compound to produce a series of concentrations in acetone. These dilutions were applied in the same manner as above to produce a range of mortality values to calculate LD_50_. Prism 8.4.3 was used to analyze dose response mortality data in best-fit sigmoidal plots with the minimum and maximum constrained to 0 and 100%, respectively [27].

### 3.5. Radical Scavenging Activity

#### 3.5.1. DPPH (2, 2-diphenyl-1-picrylhydrazyl) Scavenging Activity

The free radical scavenging activity of compounds **1**–**5** was determined based on the scavenging activity of stable DPPH, as described by Mothana et al. (2019) [28]. Seven concentrations of each sample (25, 50, 100, 500, 1000 μg/mL) were prepared by mixing with 0.125 mL of 0.2 mM methanol solution of DPPH. The negative control was one mL of methanol. An ascorbic acid positive control was prepared at the same concentrations as the test samples (Table 2). Absorbance was measured at λ = 517 nm after 30 min of incubation in the dark. DPPH percent inhibition of antioxidant effect was calculated using the formula:

% of anti-radicle activity = [(Abs _control_ − Abs _sample_)/Abs _control_] × 100

Assays were run in triplicate and means and standard errors were calculated.

#### 3.5.2. ABTS^•+^ Radical Cation Scavenging Activity

Antioxidative activity of compounds **1**–**5** was also resolved utilizing the 2,2′-azino-bis (3-ethylbenzothiazoline-6-sulfonate radical cation (ABTS^•+^**)** method, as reported by Alqahtani et al., 2019 [29], with minor modification. In brief, ABTS and potassium persulfate were prepared in deionized water to a 7 and 2.45 millimolar final concentration. To a 50-μg/mL ABTS concentration, various concentration of each extract was pipetted (1:1) and the absorbance reading (λ_734_ nm) was taken after 1 h of reaction initiation using UV-vis spectrophotometer. The capacity of each extract to exert antioxidant was determined based on the absorbance of ABTS reduced solution according to the following formula:

% of radical scavenging activity = [(Abs _control_ − Abs _sample_)/Abs _control_] × 100

## 4. Conclusions

Isolation of secondary metabolites from the dichloromethane and *n*-butanol extracts of *N. oppositifolia* led to the identification of two flavonoids (compounds **1** and **2**) and three phenylethanoid glycosides (compounds **3**–**5**). This study is the first to report the insecticidal and free radical scavenging activities of compounds from genus, *Nuxia,* including *N. oppositifolia*. The results indicate that further phytochemical and biological examination of this plant should be conducted with the aim of developing new classes of environmentally-friendly insecticidal agents. In addition, *N. oppositifolia* appears to be a rich source of natural antioxidant phenolics.

## Figures and Tables

**Figure 1:**
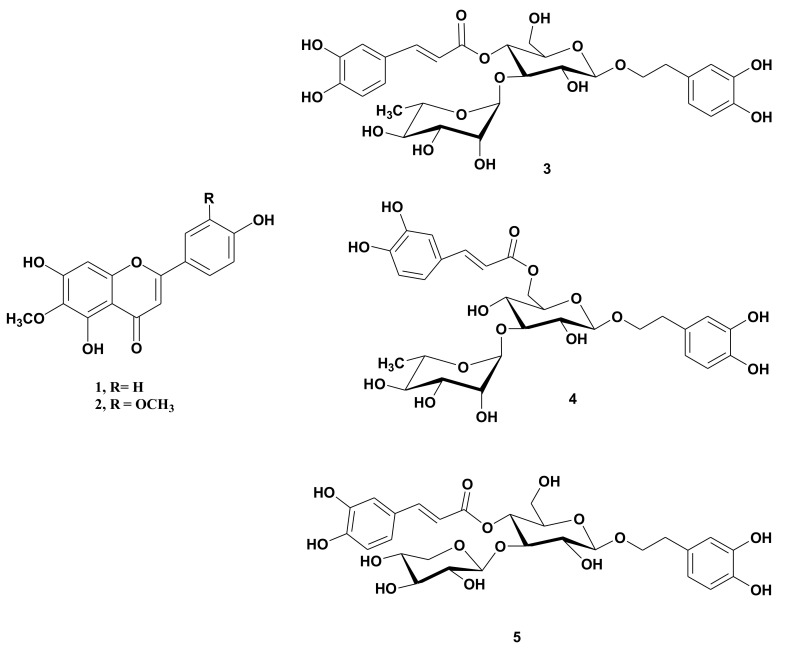
Chemical structures of the isolated compounds (**1**–**5**) from *N. oppositifolia*.

**Table 1:** Percent mortality of *N. oppositifolia* isolated compounds (**1**,**2**,**6**–**16**) at 24 h using the ORL1952 strain of *Ae. aegypti* (*n* = 3).

Compounds	Adulticidal Activity		
	Average Percent Mortality 5 μg/mosquito	LD_50_ (95% CI) * (μg/mosquito)	R^2^
Hispidulin (**1**)	70.0 ± 26.5	-	-
Jaceosidin (**2**)	63.3 ± 20.8	-	-
β-sitostosterol (**6**)	96.7 ± 5.8	2.10 (1.90–2.09)	0.9820
Katononic acid (**7**)	90.0 ± 10.0	2.22 (1.89–2.53)	0.9424
3,4-Seco olean-12-en-3,30-dioic acid (**8**)	86.7 ± 5.8	2.08 (n.d.*)	0.7976
Oleanolic acid (**9**)	86.7 ± 23.1	2.15 (1.64-2.67)	0.8642
3-Oxolupenal (**10**)	76.7 ± 5.8	-	-
Ifflaionic acid (**11**)	73.3 ± 37.9	-	-
Ursolic acid (**12**)	73.3 ± 20.8	-	-
3-Oxolupenol (**13**)	70.0 ± 10.0	-	-
Plectranthoic acid (**14**)	63.3 ± 5.8	-	-
Maslinic acid (**15**)	66.7 ± 15.3	-	-
Asiatic acid (**16**)	56.7 ± 15.3	-	-
Untreated	0	-	-
Solvent control (acetone only)	0	-	-
0.15 ng permethrin	37.0 ± 6.0	-	-
0.23 ng permethrin	63.0 ± 5.0	-	-
2.37 ng permethrin	100 ± 0	-	-

* After the primary screening of the compounds, compounds showing mortality ≥80% were further assessed for LD_50_ in dose-response bioassays. LD_50_ and 95% confidence intervals were calculated using Prism 8.4.3. Data for compound **8** did not allow to accurately calculate 95% CI, and so it is not determined (n.d.).

**Table 2:** Free radical scavenging activity of *N. oppositifolia* isolated compounds (**1**–**5**).

Sample		DPPH-Radical Scavenging Activity in %				
	25	50	100	500	1000	IC_50_
(µg/mL)						
Hispidulin (**1**)	45.7 ± 0.3	56.9 ± 0.2	67.9 ± 0.1	72.5 ± 0.4	79.2 ± 0.1	34.5 ± 0.8
Jaceosidin (**2**)	44.5 ± 0.3	53.7 ± 0.1	58.4 ± 0.2	71.3 ± 0.2	79.8 ± 0.1	39.8 ± 0.4
Verbascoside (**3**)	42.3 ± 2.4	57.3 ± 0.7	64.7 ± 0.2	67.9 ± 0.1	79.2 ± 0.7	37.9 ± 1.6
Isoverbascoside (**4**)	42.3 ± 0.6	44.8 ± 1.6	59.1 ± 0.5	70.5 ± 0.6	75.7 ± 0.4	68.1 ± 1.7
Conandroside (**5**)	49.3 ± 0.2	55.8 ± 1.9	65.5 ± 0.5	71.8 ± 0.2	79.8 ± 1.2	27.3 ± 1.1
l-Ascorbic acid	80.7 ± 2.0	85.1 ± 1.3	85 ± 1.2	88.7 ± 2.4	90.7 ± 1.4	5.0 ± 0.15
	(ABTS^•+^) **Radical Cation Scavenging Activity in %**					
Hispidulin (**1**)	47.7 ± 2.7	55.3 ± 0.7	64.4 ± 0.5	69.5 ± 0.7	75.7 ± 1.7	32.4 ± 2.7
Jaceosidin (**2**)	49.2 ± 0.5	56.3 ± 1	63.1 ± 0.4	70.6 ± 0.5	77.1 ± 0.1	28.0 ± 0.8
Verbascoside (**3**)	43.7 ± 1.2	49.3 ± 0.5	54.6 ± 0.5	63.1 ± 0.7	76.5 ± 0.4	58.5 ± 5.4
Isoverbascoside (**4**)	44.2 ± 0.7	51.4 ± 0.9	51.4 ± 1.9	66.9 ± 0.5	73.7 ± 0.3	45.5 ± 2.6
Conandroside (**5**)	47.4 ± 1.5	52.3 ± 0.2	60.6 ± 0.7	65.3 ± 0.9	75.8 ± 0.2	38.2 ± 3.8
l-Ascorbic acid	80.7 ± 2.4	81.2 ± 2.1	84.2 ± 1.9	87.2 ± 2.4	88.7 ± 2.1	6.0 ± 0.85

*p* < 0.005, Data results were presented as means of variability % ± standard deviation (*n* = 3).

## Supplementary Materials

Spectral data of known compounds (**1**–**3**, **5**) are available online.

## Sample Availability

Samples of the compounds are available from the authors.
