# Supplementary material for: Insecticidal Activity and Free Radical Scavenging Properties of Isolated Phytoconstituents from the Saudi Plant Nuxia oppositifolia (Hochst.)
Source: Molecules. 2021 Feb 9;26(4):914. doi: 10.3390/molecules26040914 (PMC7915531; doi:10.3390/molecules26040914)
Supplement: Supplementary file 1 [file molecules-26-00914-s001.zip › Adulticidal Suplemental data for_Nuxia.pdf]

USDA ARS CMAVE
Mosquito & Fly Research Unit
Estep Laboratory
08NOV2020

Calculation verification of Nuxia LD50 data from 2016 testing

Data location: USDA ARS CMAVE Notebook: JL-8, pages 40-42

Percent Mortality Counts:

log(ug/mosq)	Compound **7**	Compound **8**	Compound **9**	N15H-12	assay
0.69897	80	60	60	90	1
-0.30103	0	0	0	0	1
-1.30103	0	0	0	0	1
-2.30103	0	0	0	0	1
0.69897	100	100	100	100	2
0.60206	90	90	90	100	2
0.477121	50	80	50	100	2
0.30103	50	20	20	40	2
0	0	0	0	0	2
-0.30103	10	0	0	0	2
0.69897			100		3
0.60206	100	90	100	100	3
0.544068	100				3
0.477121	70	80	100	90	3
0.39794		100		100	3
0.30103	40	10	70	50	3
0.176091				20	3

0	10	50	10	0	3
-0.30103	0	10	0		3

Rerun analysis (on 08NOV2020) using data above in Prism v8.4.3 to check:

Compounds	LD50 (95% CI)	R2
Compound **6**	2.10 (1.90-2.09)	0.9820
Compound **7**	2.22 (1.89-2.53)	0.9424
Compound **8**	2.08 (wide)	0.7976
Compound **9**	2.15 (1.64-2.67)	0.8642

	Compound **6**	Compound **7**	Compound **8**	Compound **9**
log(agonist) vs. response -- Variable slope (four parameters)			Ambiguous	
Best-fit values				
Bottom	= 0.000	= 0.000	= 0.000	= 0.000
Top	= 100.0	= 100.0	= 100.0	= 100.0
LogEC50	0.3029	0.3457	~ 0.3175	0.3314
HillSlope	9.048	3.361	~ 45.83	3.297
EC50	2.009	2.217	~ 2.077	2.145
Span	= 100.0	= 100.0	= 100.0	= 100.0
Std. Error				
LogEC50	0.008185	0.02863	~ 1.520	0.04887
HillSlope	1.946	0.6445	~ 4239	1.052
95% CI (profile likelihood)				
LogEC50	0.2793 to 0.3203	0.2772 to 0.4035	(Very wide)	0.2159 to 0.4257
HillSlope	5.682 to ???	2.290 to 5.071	(Very wide)	1.800 to 8.937
EC50	1.902 to 2.091	1.893 to 2.532	(Very wide)	1.644 to 2.665
Goodness of Fit				
Degrees of Freedom	14	14	14	14
R squared	0.9820	0.9424	0.7976	0.8642
Sum of Squares	569.1	1474	5250	3934
Sy.x	6.376	10.26	19.36	16.76
Constraints				
Bottom	Bottom = 0	Bottom = 0	Bottom = 0	Bottom = 0
Top	Top = 100	Top = 100	Top = 100	Top = 100
Number of points				

# of X values	18	19	19	19
# Y values analyzed	16	16	16	16
