# Supplementary material for: Insecticidal Activity and Free Radical Scavenging Properties of Isolated Phytoconstituents from the Saudi Plant Nuxia oppositifolia (Hochst.)
Source: Molecules. 2021 Feb 9;26(4):914. doi: 10.3390/molecules26040914 (PMC7915531; doi:10.3390/molecules26040914)
Supplement: Supplementary file 1 [file molecules-26-00914-s001.zip › SUPPLEMENTARY MATERIAL_Nuxia.pdf]

SUPPLEMENTARY MATERIAL

Insecticidal Activity and Free Radical Scavenging Properties of Isolated Phytoconstituents from the Saudi Plant *Nuxia oppositifolia* (Hochst.)

Shaza M. Al-Massarani[1*], Ali A. El-Gamal[1,2], Adnan J. Al-Rehaily[1], Ebtesam S. Al-Sheddi[1], Mai M. Al-Oqail[1], Nida N. Farshori[1], Alden S. Estep[3,4], Nurhayat Tabanca[5], James J. Becnel[3]

[1]Department of Pharmacognosy, King Saud University, College of Pharmacy, P.O. Box 2457, Riyadh Saudi Arabia

[2]Department of Pharmacognosy, Mansoura University, College of Pharmacy, 35516, El-Mansoura, Egypt

[3]USDA, ARS, Center for Medical, Agricultural, and Veterinary Entomology, Gainesville, FL 32608 USA

[4]Navy Entomology Center of Excellence, CMAVE Detachment, 1700 SW 23rd Drive, Gainesville, FL 32608 USA

[5]USDA-ARS, Subtropical Horticulture Research Station, 13601 Old Cutler Rd., Miami, FL 33158 USA

*Corresponding author: salmassarani@ksu.edu.sa

Abstract

Abstract: Chromatographic purification of the alcoholic extract of the aerial parts of the Saudi plant *Nuxia oppositifolia* (Hochst.) Benth., led to the isolation of five phenolic compounds. Two flavones, hispidulin (**1**) and jaceosidin (**2**), and the phenylethanoid glycosides, verbascoside (**3**), isoverbascoside (**4**) and conandroside (**5**), were identified. Their chemical structures were determined based on extensive spectroscopic analyses. The insecticidal activity of compounds **1** and **2,** in addition to 11 compounds isolated in a previous research (**6**-**16**), was evaluated against the Yellow Fever mosquito, *Aedes aegypti*. Four compounds displayed adulticidal activity with LD_{50} values of 2–2.3 µg/mosquito. Further, the free radical scavenging properties of the plant extracts and compounds (**1-5**) were evaluated by measuring 1,1-diphenyl-2-picrylhydrazyl radical (DPPH) and 2,2′-azino-bis (3-ethylbenzothiazoline-6-sulfonate radical cation ($ABTS^{\bullet+}$) scavenging activity. All compounds exhibited notable activity, compared to the positive control, L-ascorbic acid. This study suggests that *N. oppositifolia* can be considered a promising source of secondary metabolites with lethal adulticidal effect against *Ae. aegypti*.

Keywords: *Nuxia oppositifolia*; flavonoids; phenylethanoid; triterpenes; Mosquito control; biopesticides; *Ae. aegypti*; free radical scavenging

Contents

Figure S1 ^{1}HNMR (500MHz, DMSO) spectrum of compound **1**
Figure S2 COSY (500 MHz, DMSO) spectrum of compound **1**
Figure S3 ^{13}CNMR (125MHz, DMSO) spectrum of compound **1**
Figure S4 DEPT experiment (125MHz, DMSO) spectrum of compound **1**
Figure S5 HSQC (500 MHz, DMSO) spectrum of compound **1**
Figure S6 HMBC (500 MHz, DMSO) spectrum of compound **1**

Figure S7 ^{1}HNMR (500MHz, DMSO) spectrum of compound **2**
Figure S8 COSY (500 MHz, DMSO) spectrum of compound **2**
Figure S9 ^{13}CNMR (125MHz, DMSO) spectrum of compound **2**
Figure S10 DEPT experiment (125MHz, DMSO) spectrum of compound **2**
Figure S11 HSQC (500 MHz, DMSO) spectrum of compound **2**

Figure S12 HMBC (500 MHz, DMSO) spectrum of compound **2**

Figure S13 ^{1}HNMR (500MHz, CD3OD) spectrum of compound **3**
Figure S14 COSY (500 MHz, CD3OD) spectrum of compound **3**
Figure S15 ^{13}CNMR (125MHz, CD3OD) spectrum of compound **3**
Figure S16 DEPT experiment (125MHz, CD3OD) spectrum of compound **3**
Figure S17 HSQC (500 MHz, CD3OD) spectrum of compound **3**
Figure S18 HMBC (500 MHz, CD3OD) spectrum of compound **3**

Figure S19 ^{1}HNMR (500MHz, CD3OD) spectrum of compound **4**
Figure S20 Expanded ^{1}HNMR (500MHz, CD3OD) spectrum of compound **4**
Figure S21 ^{13}CNMR (125MHz, CD3OD) spectrum of compound **4**
Figure S22 DEPT experiment (125MHz, CD3OD) spectrum of compound **4**

Figure S19 ^{1}HNMR (500MHz, CD3OD) spectrum of compound **5**
Figure S20 COSY (500 MHz, CD3OD) spectrum of compound **5**
Figure S21 ^{13}CNMR (125MHz, CD3OD) spectrum of compound **5**
Figure S22 DEPT experiment (125MHz, CD3OD) spectrum of compound **5**
Figure S23 HSQC (500 MHz, CD3OD) spectrum of compound **5**
Figure S24 HMBC (500 MHz, CD3OD) spectrum of compound **5**

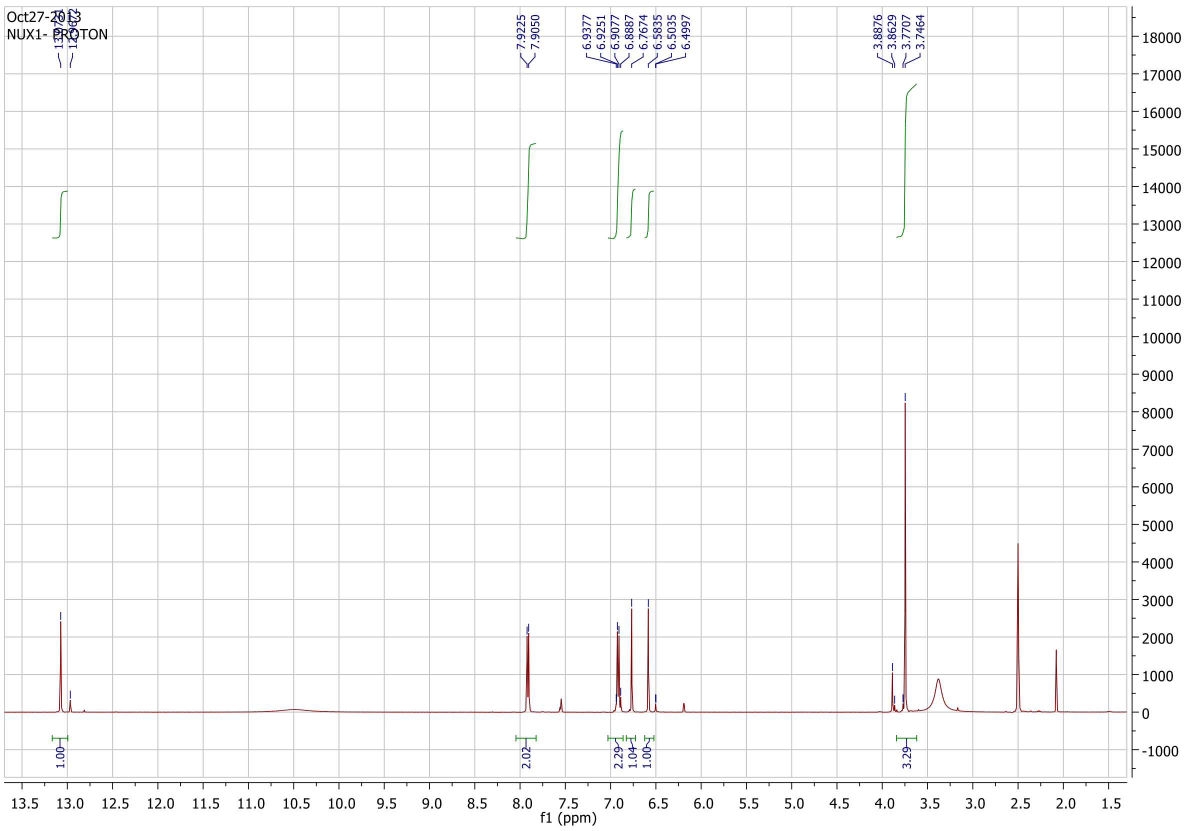

Figure S1 ^{1}HNMR (500MHz, CD_3OD) spectrum of compound **1**

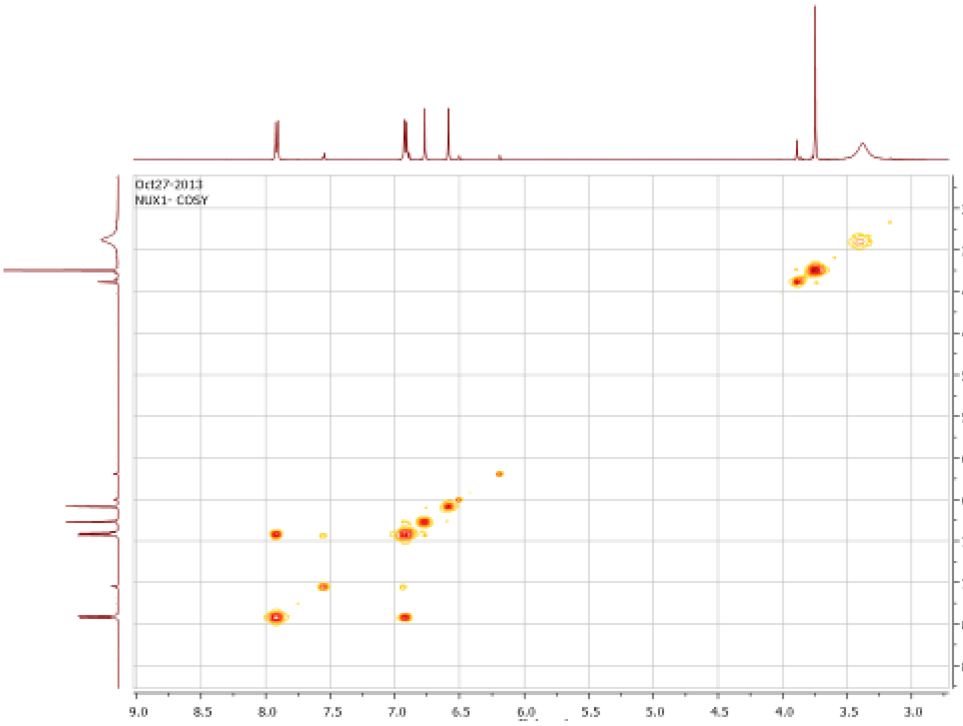

Figure S2 COSY (500 MHz, CD_3OD) spectrum of compound **1**

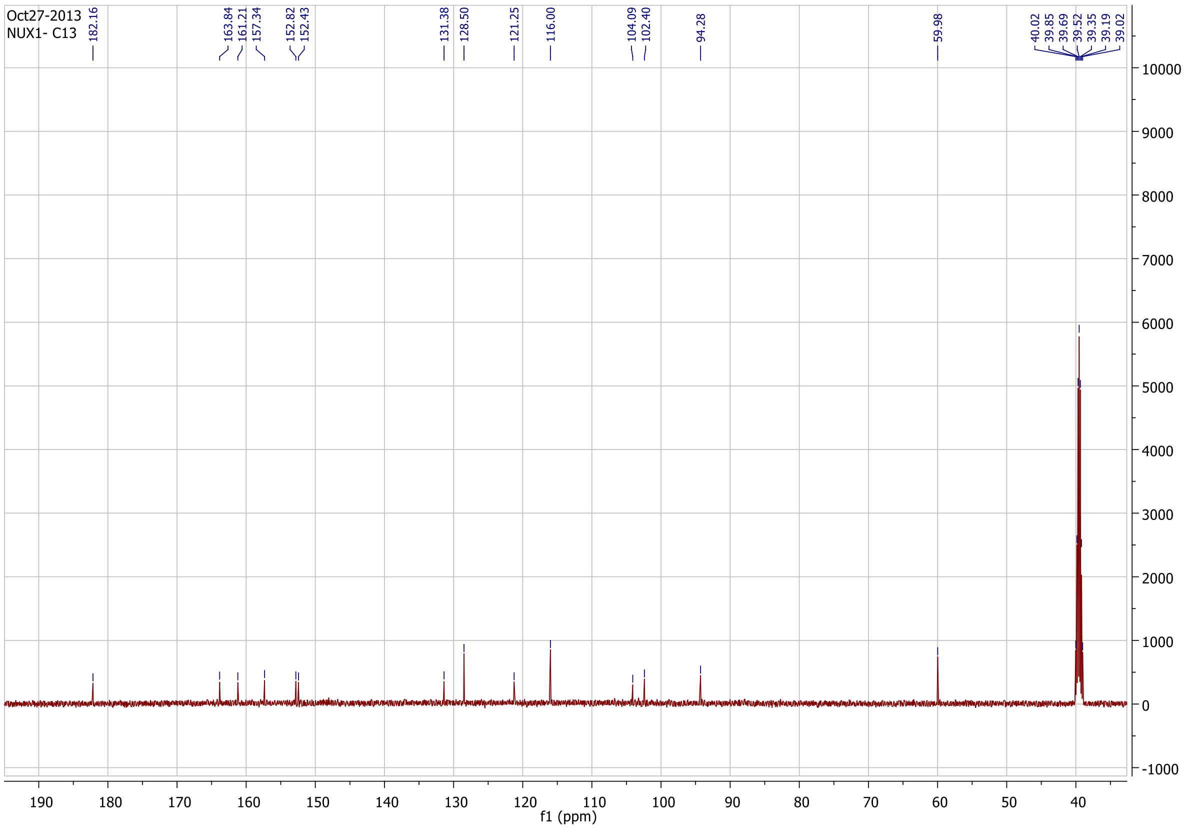

Figure S3 ^{13}CNMR (125MHz, CD_3OD) spectrum of compound **1**

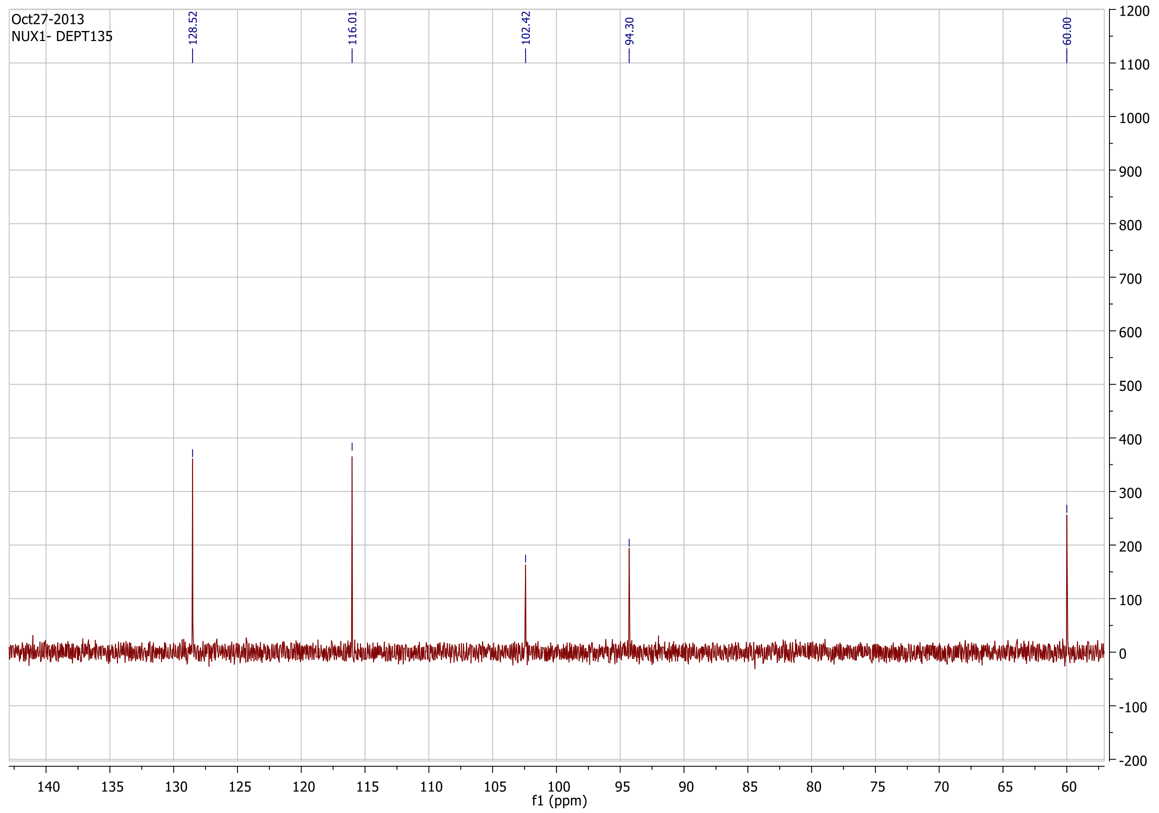

Figure S4 DEPT experiment (125MHz, CD_3OD) spectrum of compound **1**

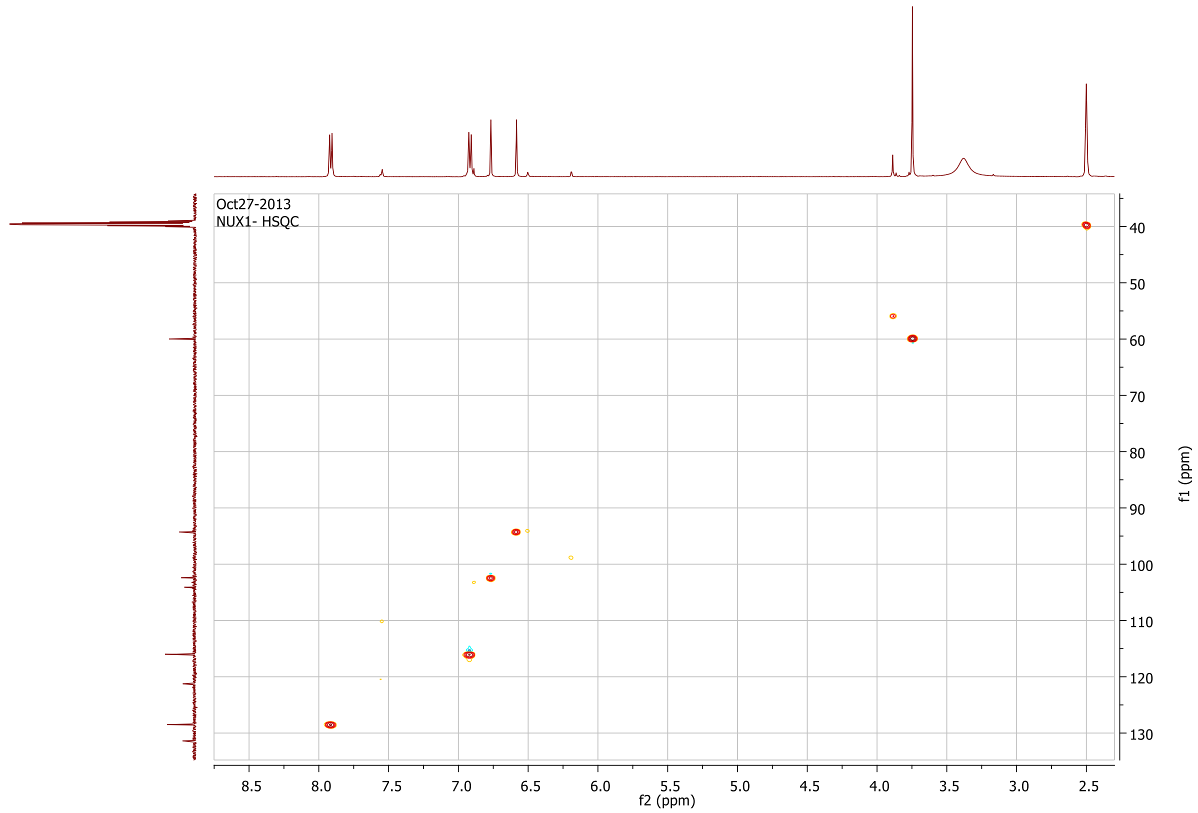

Figure S5 HSQC (500 MHz, CD_3OD) spectrum of compound **1**

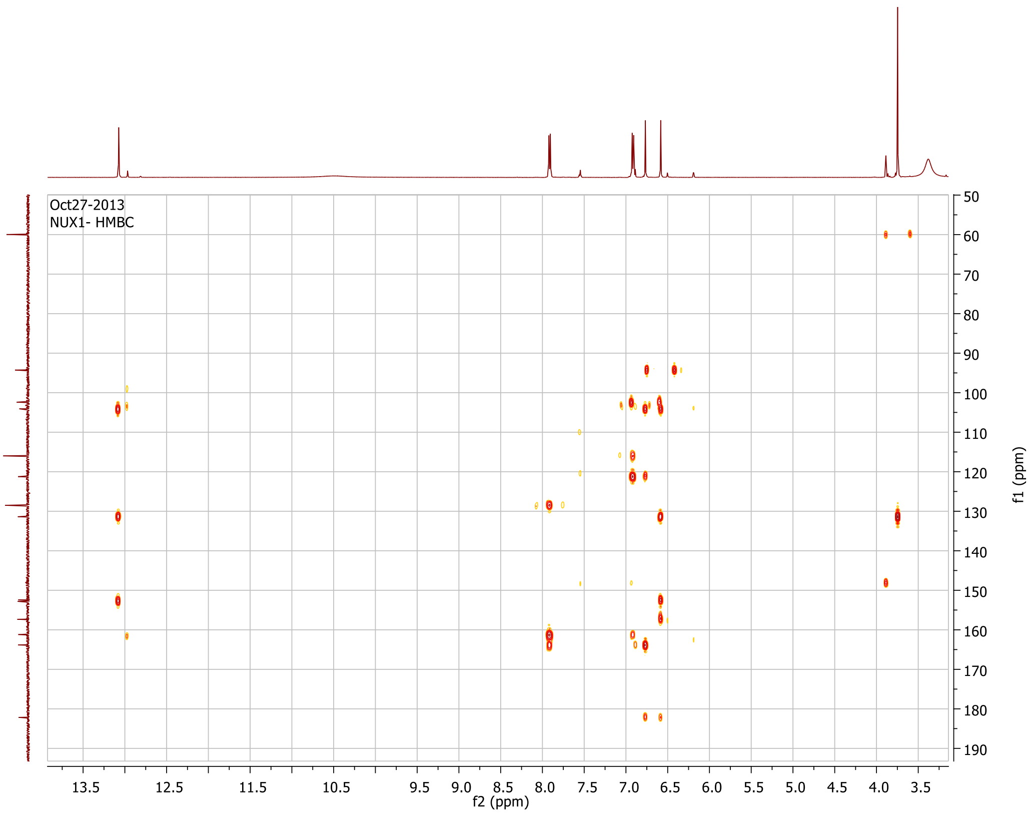

Figure S6 HMBC spectrum (500 MHz, CD3OD) spectrum of compound **1**

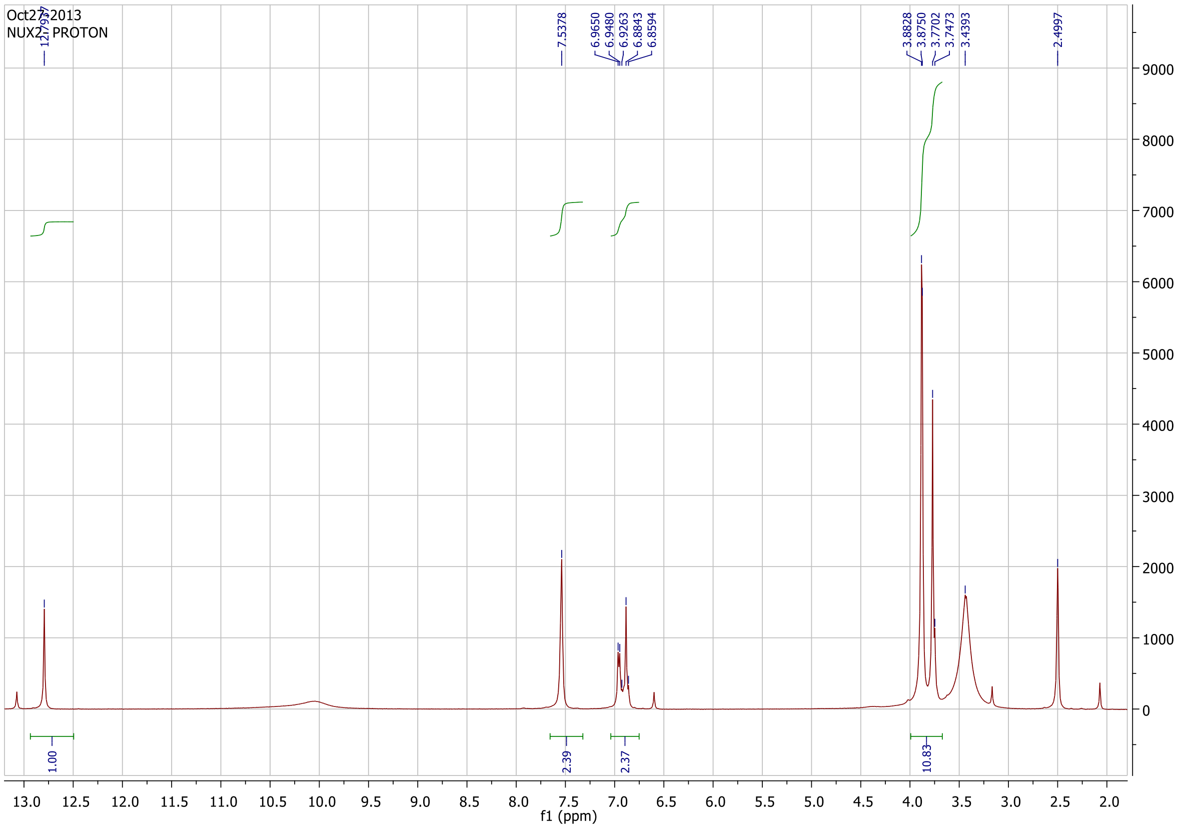

Figure S7 ^{1}HNMR (500MHz, CD3OD) spectrum of compound **2**

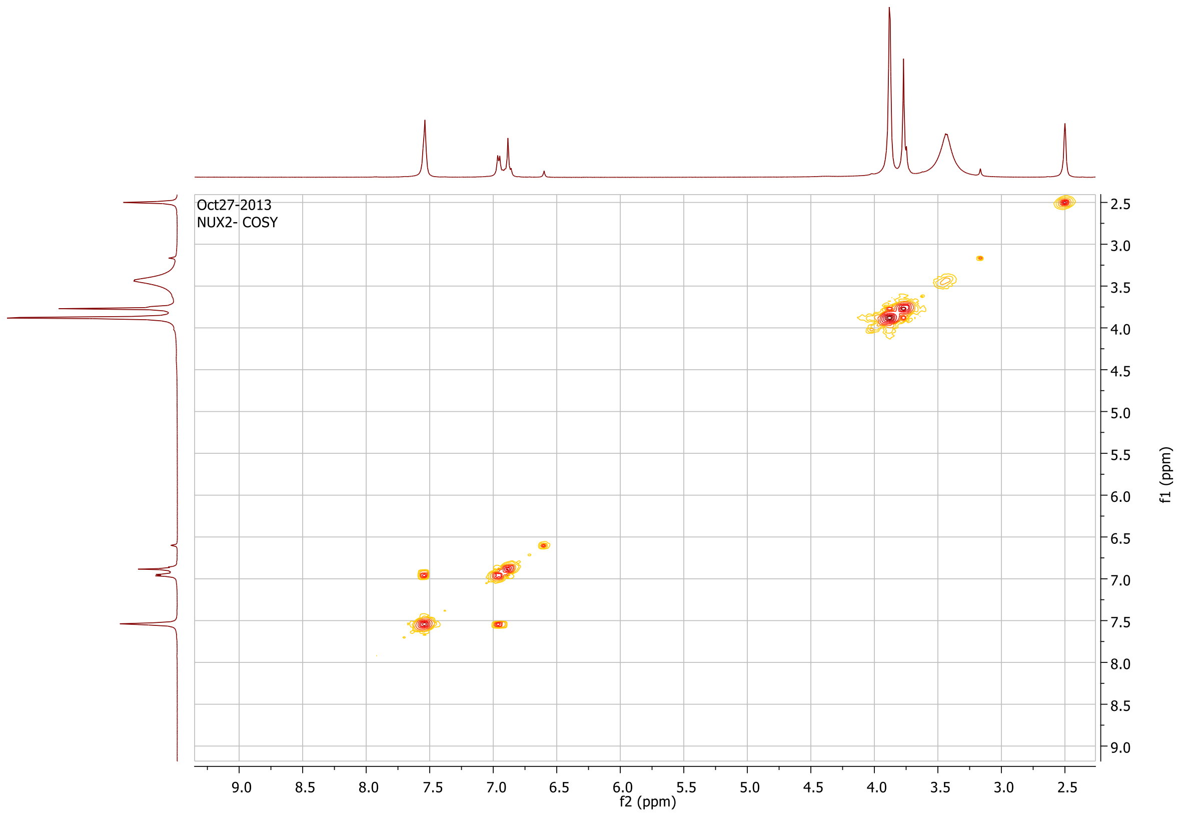

Figure S8 COSY (500 MHz, CD_3OD) spectrum of compound **2**

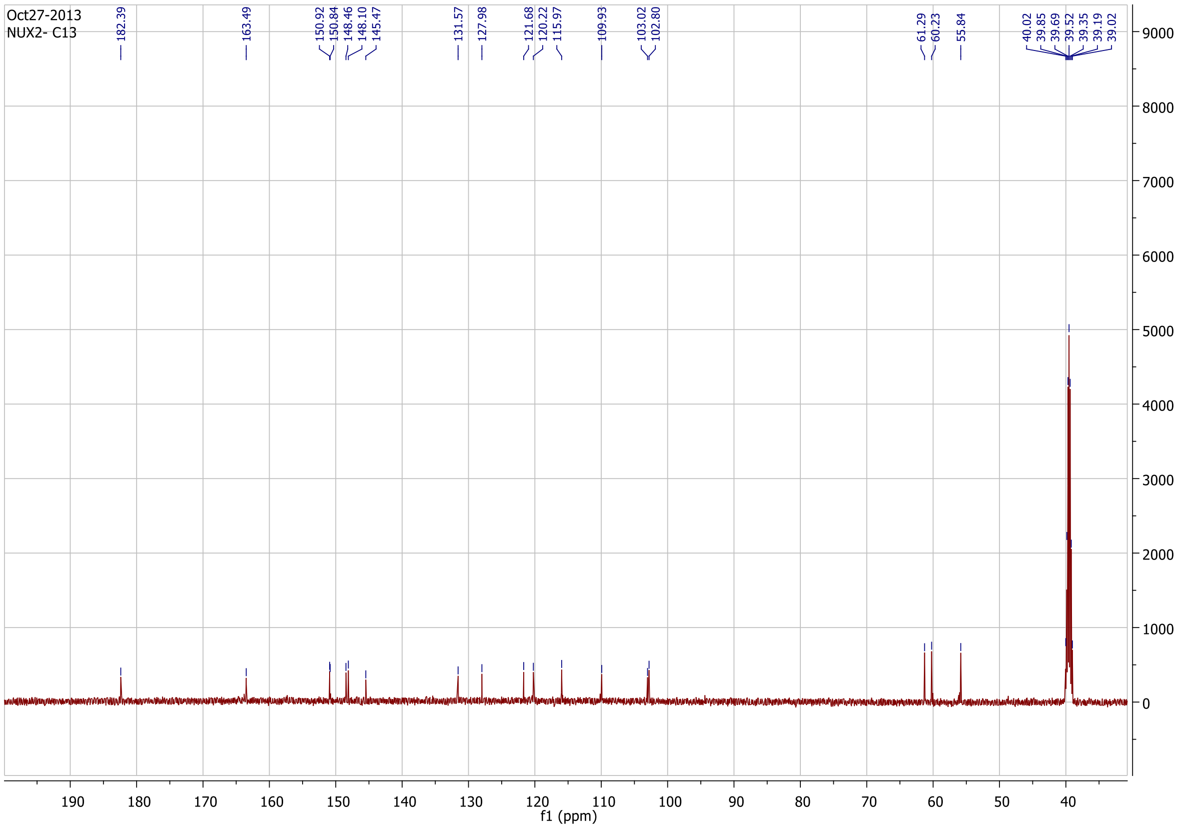

Figure S9 ^{13}CNMR (125MHz, CD_3OD) spectrum of compound **2**

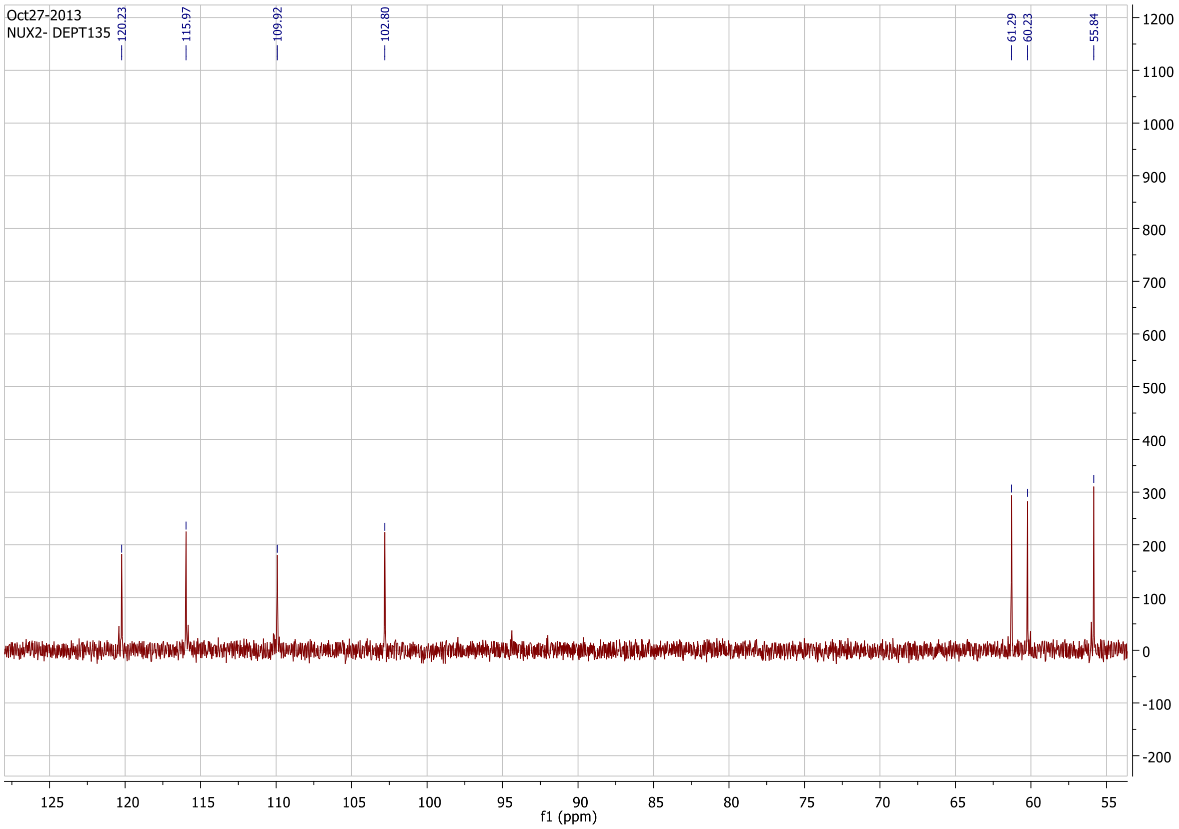

Figure S10 DEPT experiment (125MHz, CD_3OD) spectrum of compound **2**

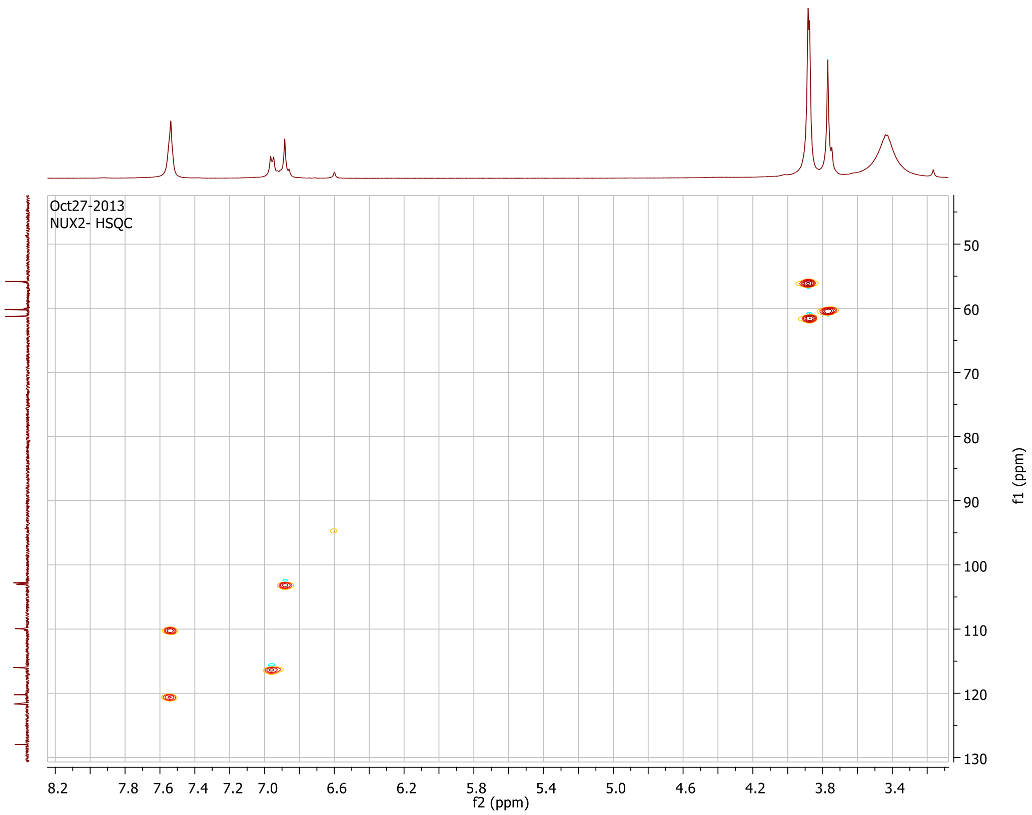

Figure S11 HSQC (500 MHz, CD_3OD) spectrum of compound **2**

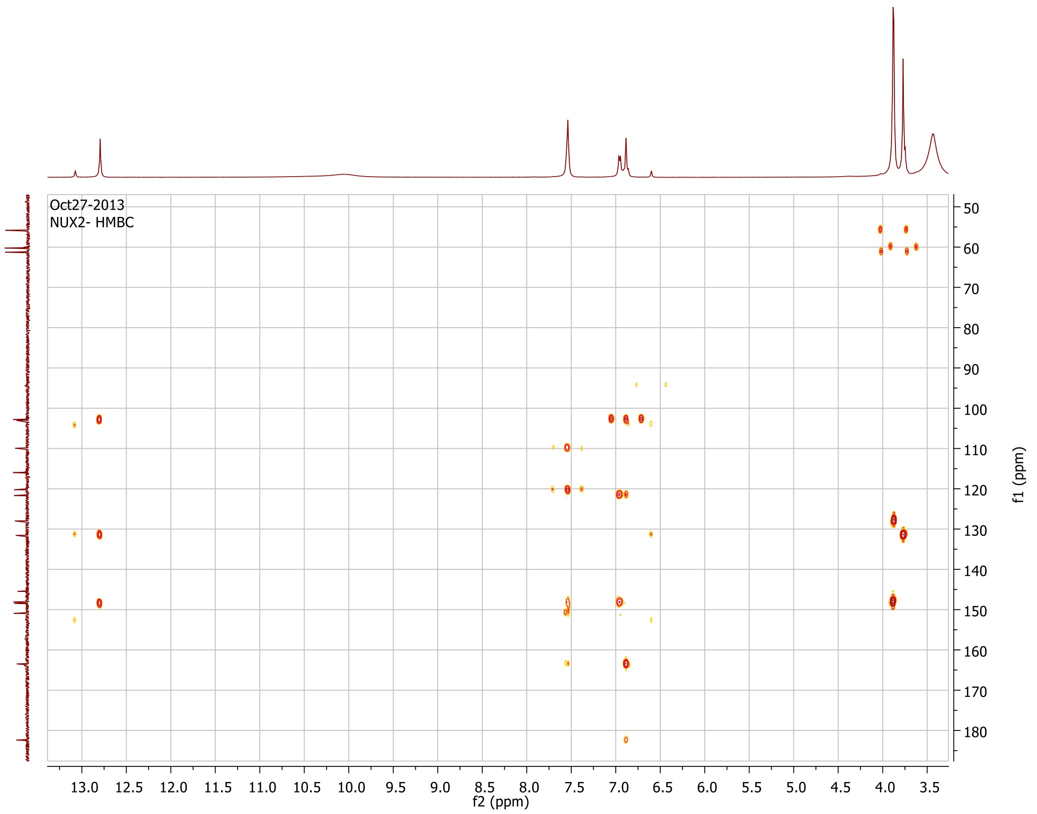

Figure S12 HMBC spectrum (500 MHz, CD_3OD) spectrum of compound **2**

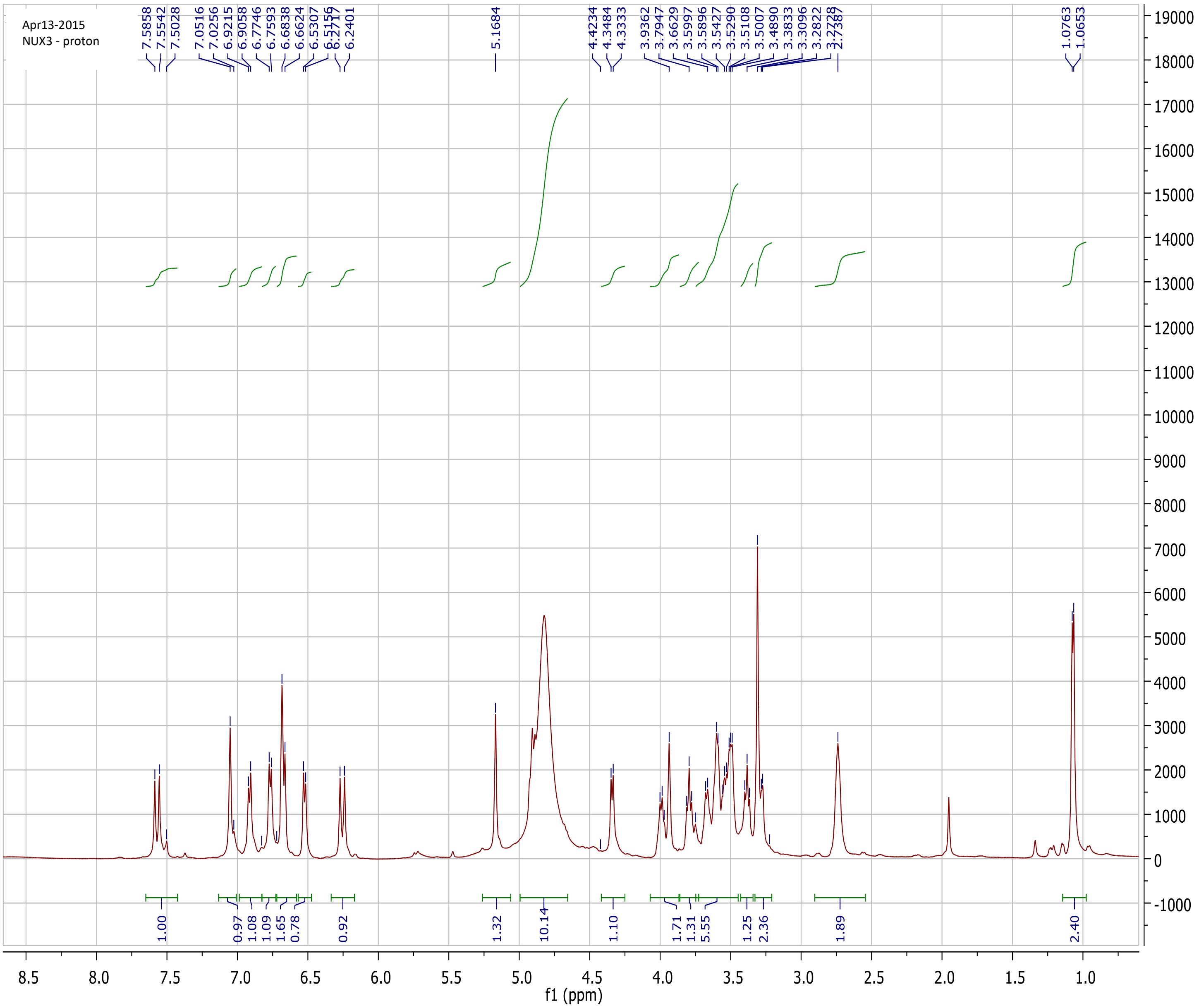

Figure S13 ^{1}HNMR (500MHz, CD_3OD) spectrum of compound **3**

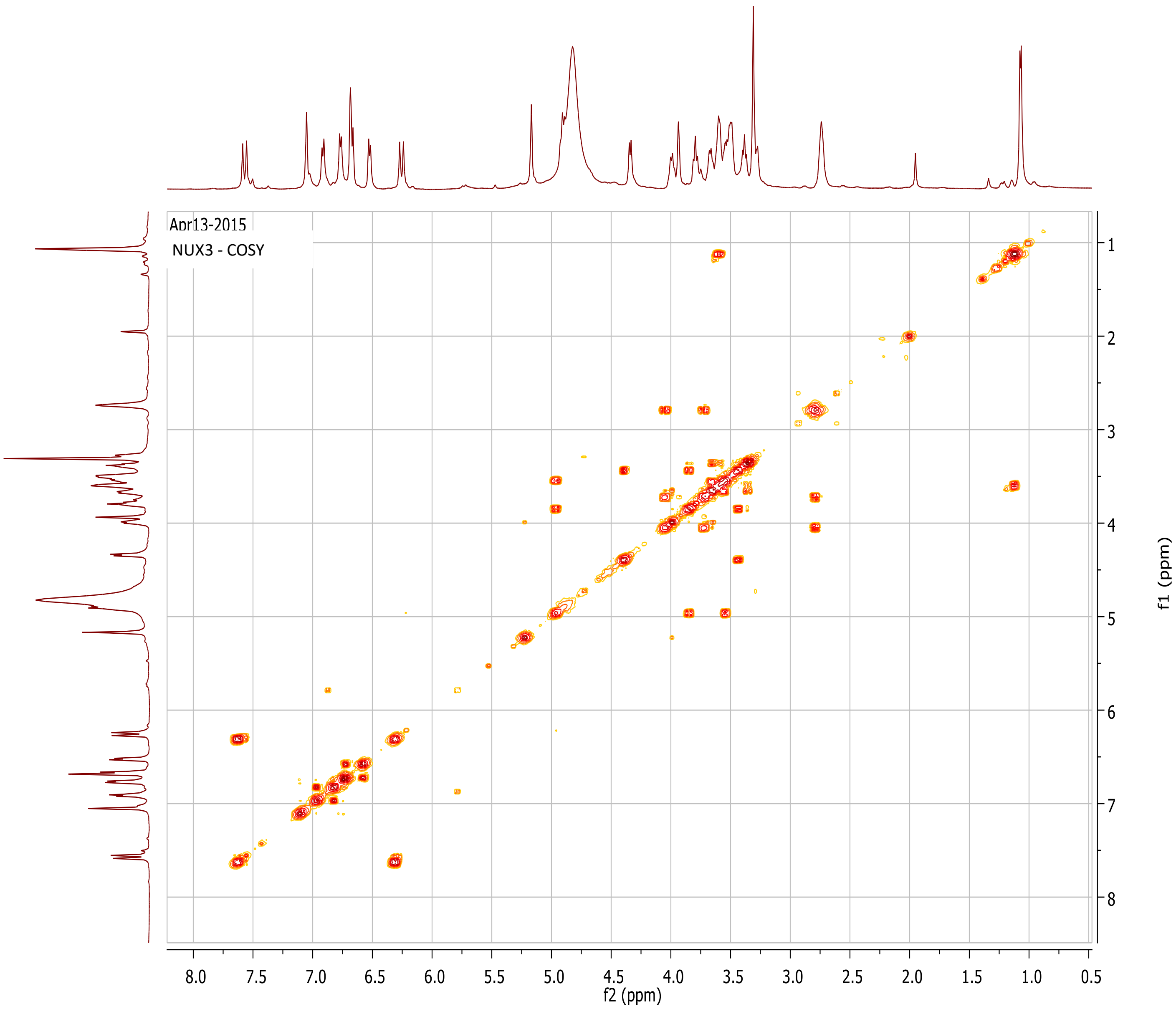

Figure S14 COSY spectrum (500 MHz, CD_3OD) spectrum of compound **3**

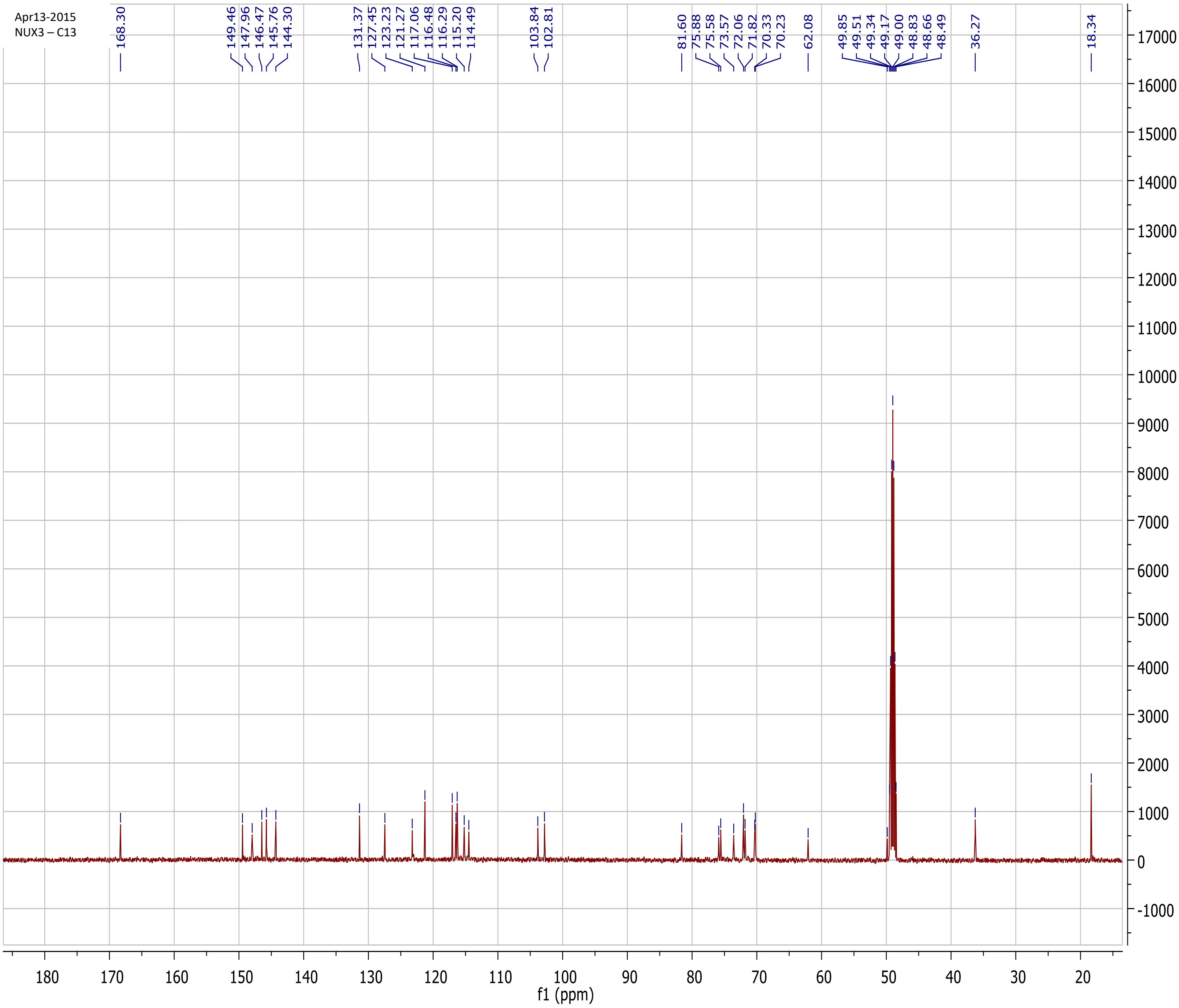

Figure S15 ^{13}CNMR (125MHz, CD_3OD) spectrum of compound **3**

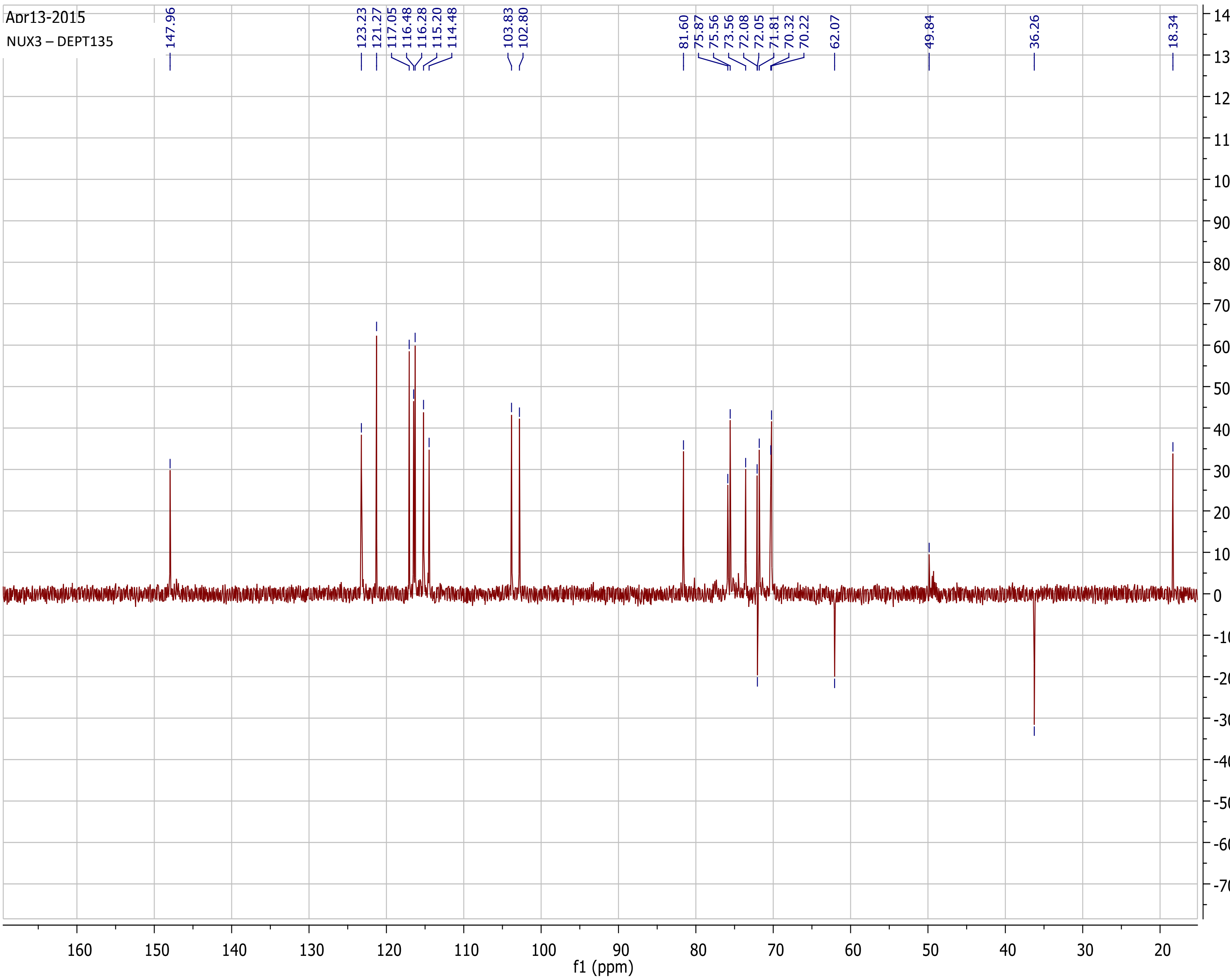

Figure S16 DEPT experiment (125MHz, CD_3OD) spectrum of compound **3**

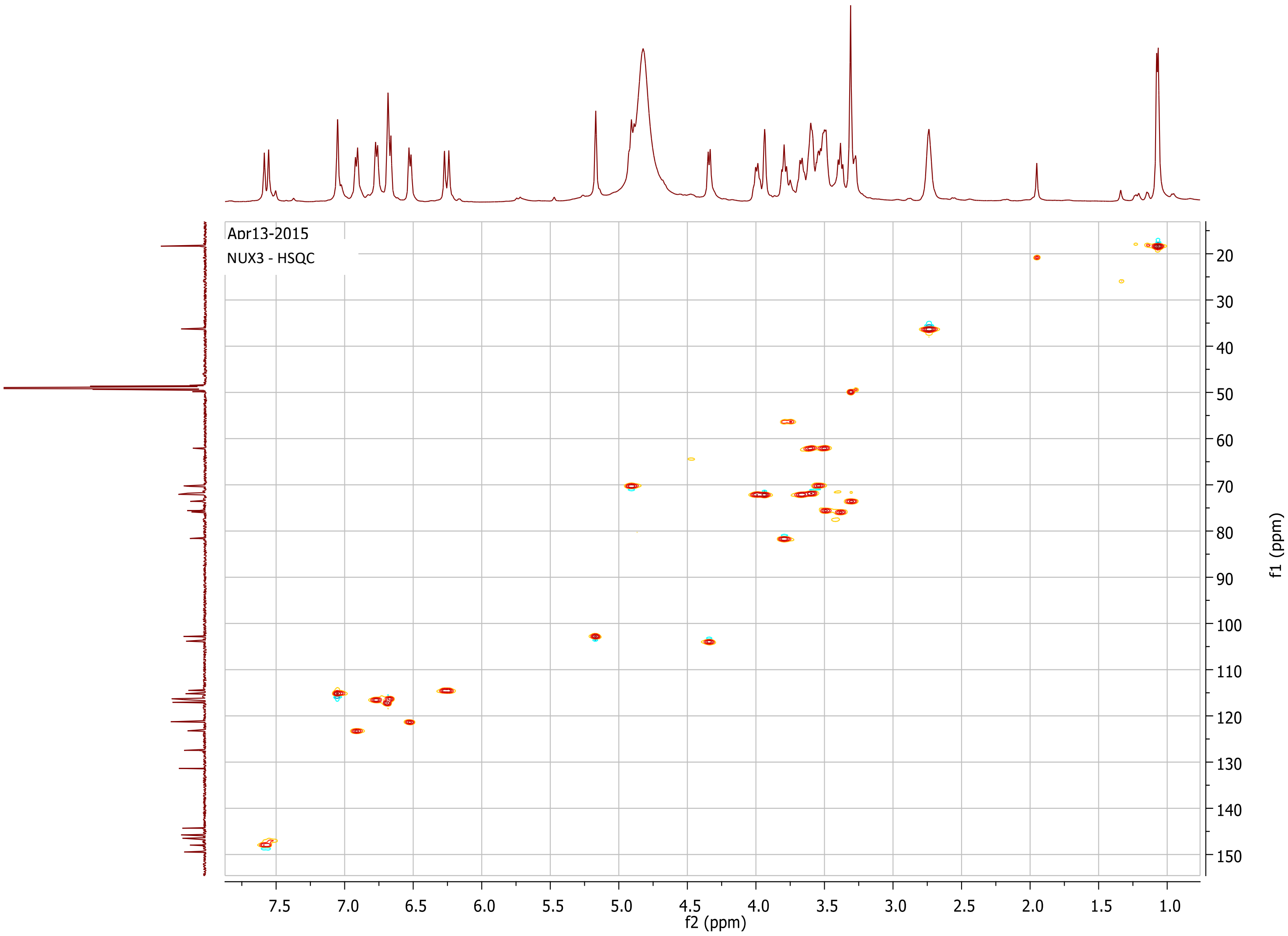

Figure S17 HSQC (500 MHz, CD3OD) spectrum of compound **3**

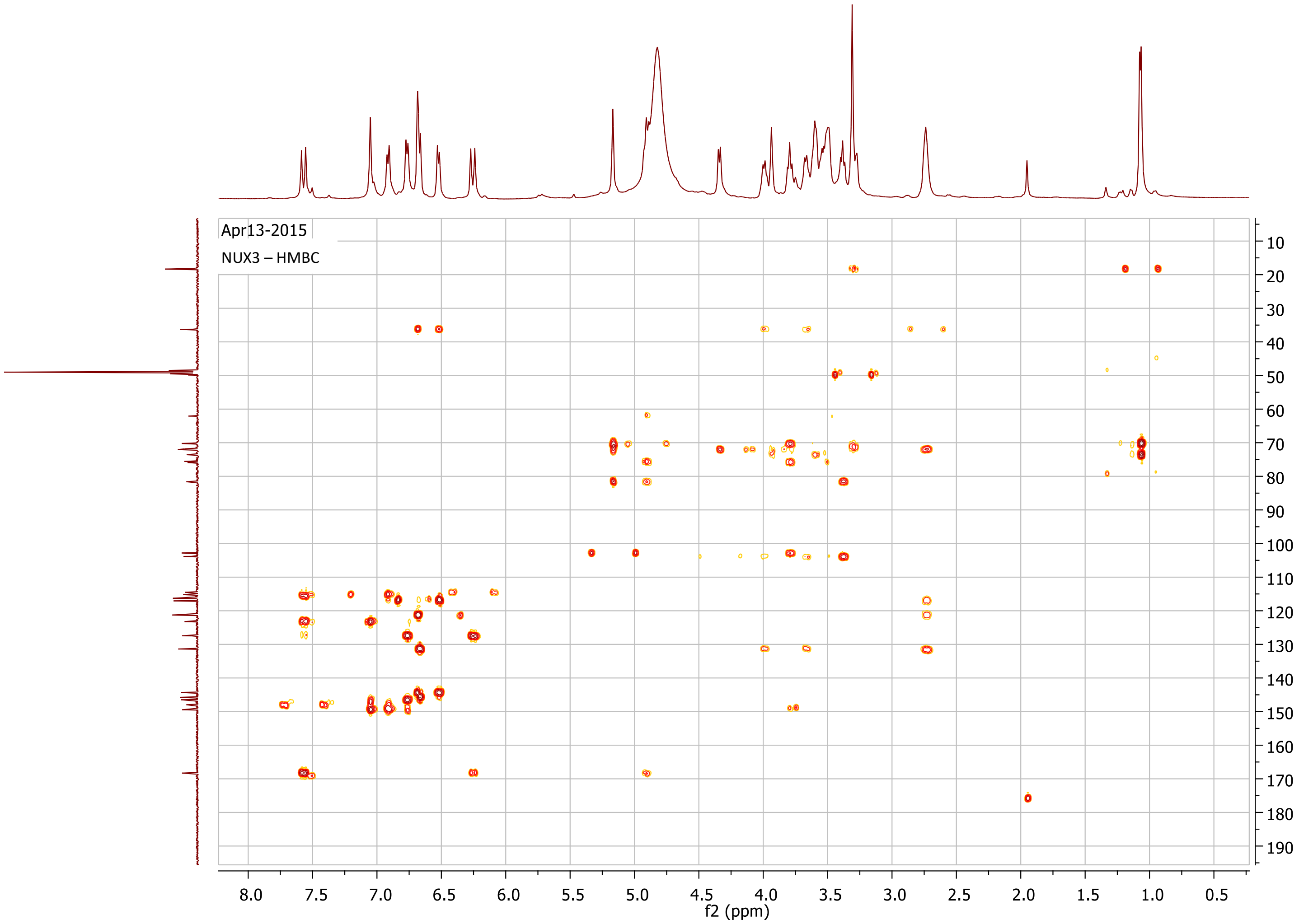

Figure S18 HMBC (500 MHz, CD_3OD) spectrum of compound **3**

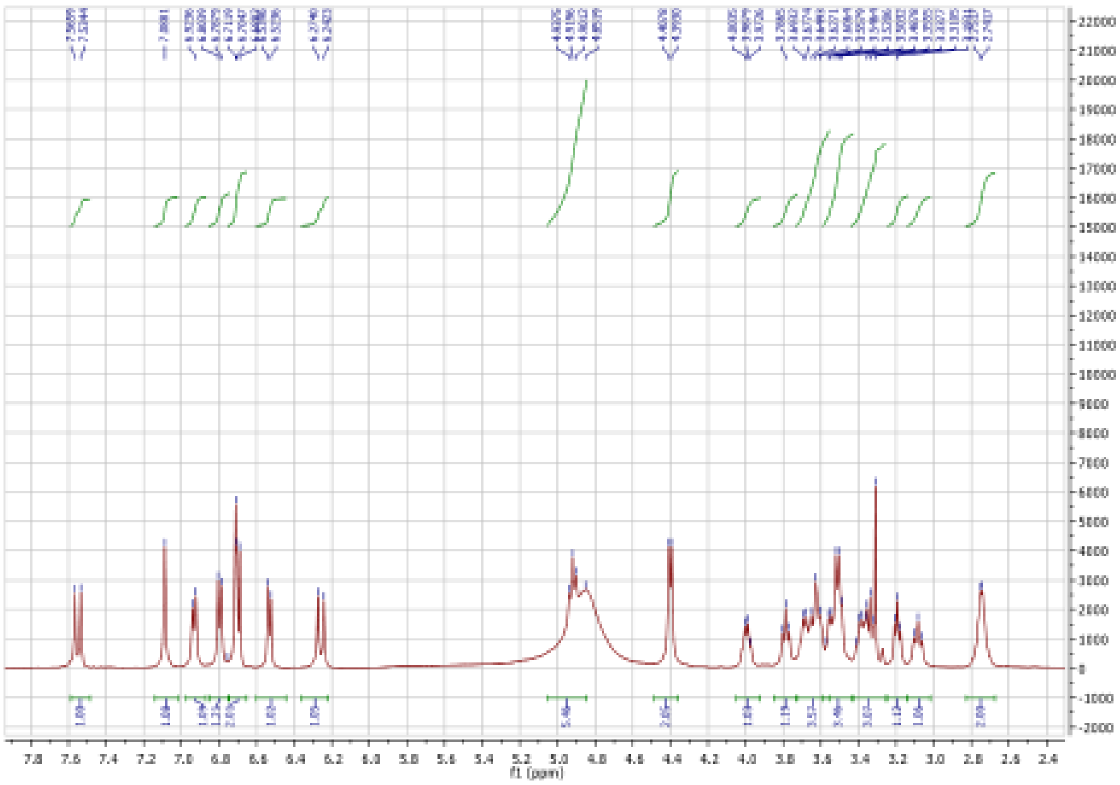

Figure S19 ^{1}HNMR (500MHz, CD_3OD) spectrum of compound **5**

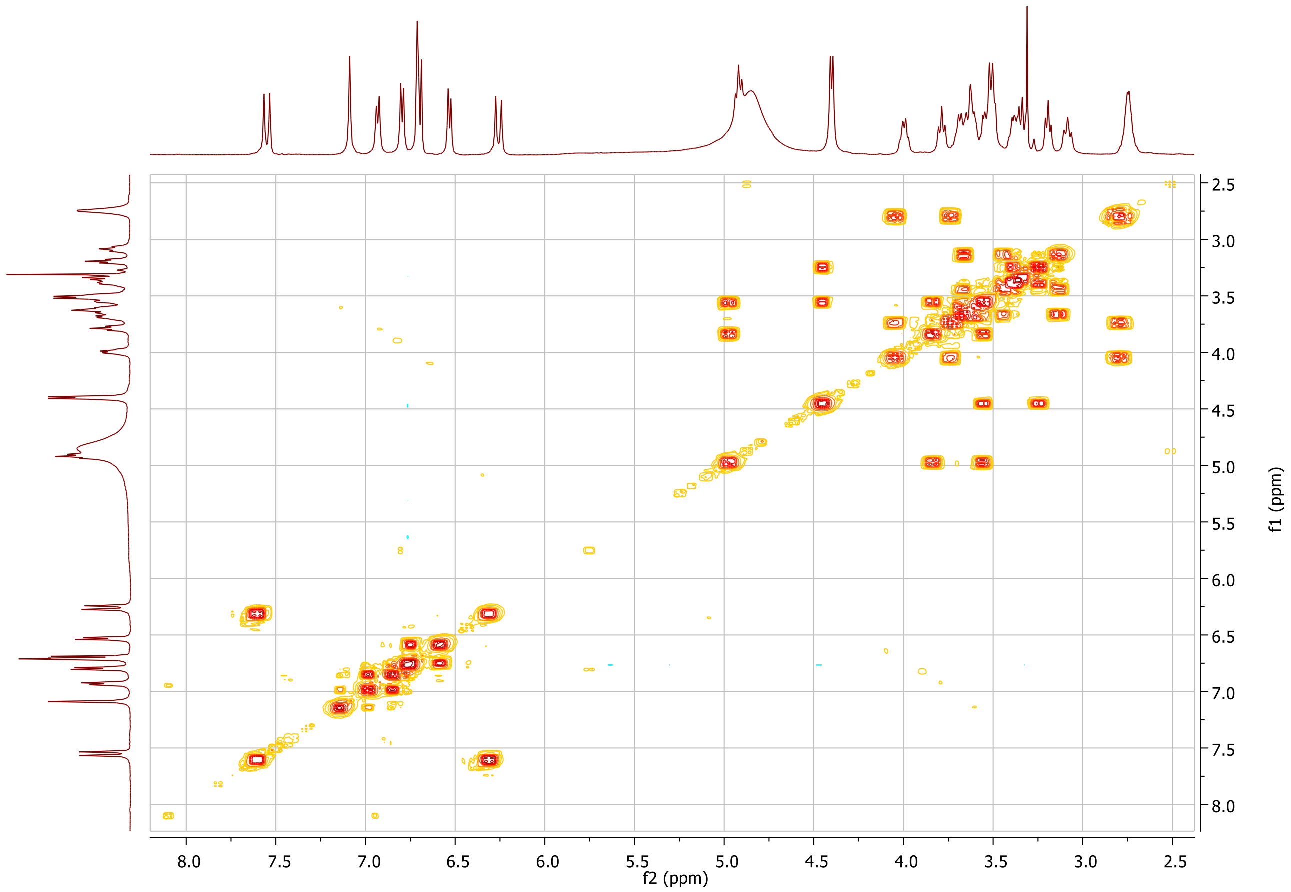

Figure S20 COSY (500 MHz, CD_3OD) spectrum of compound **5**

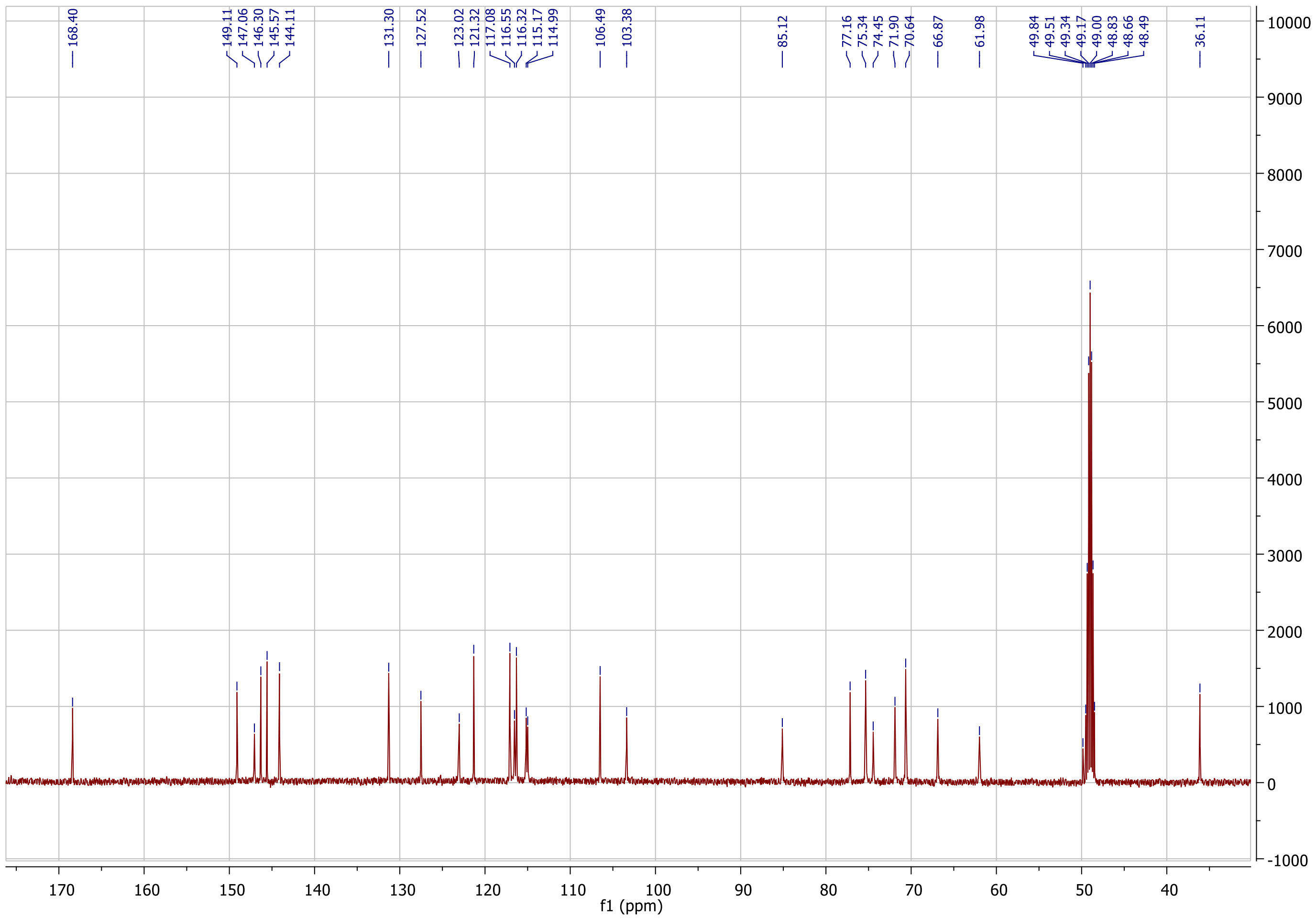

Figure S21 ^{13}CNMR (125MHz, CD_3OD) spectrum of compound **5**

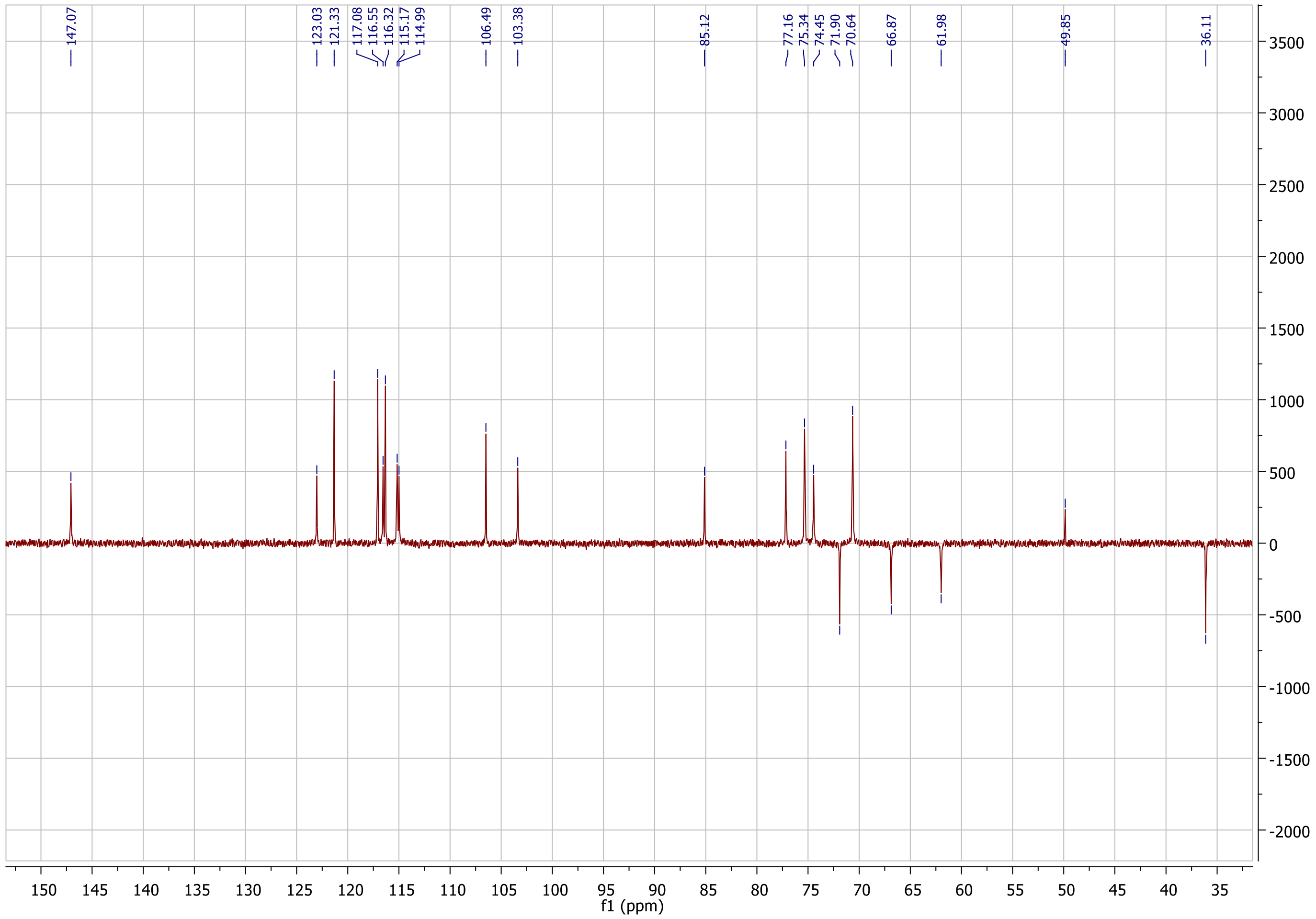

Figure S22 DEPT experiment (500 MHz, CD_3OD) spectrum of compound **5**

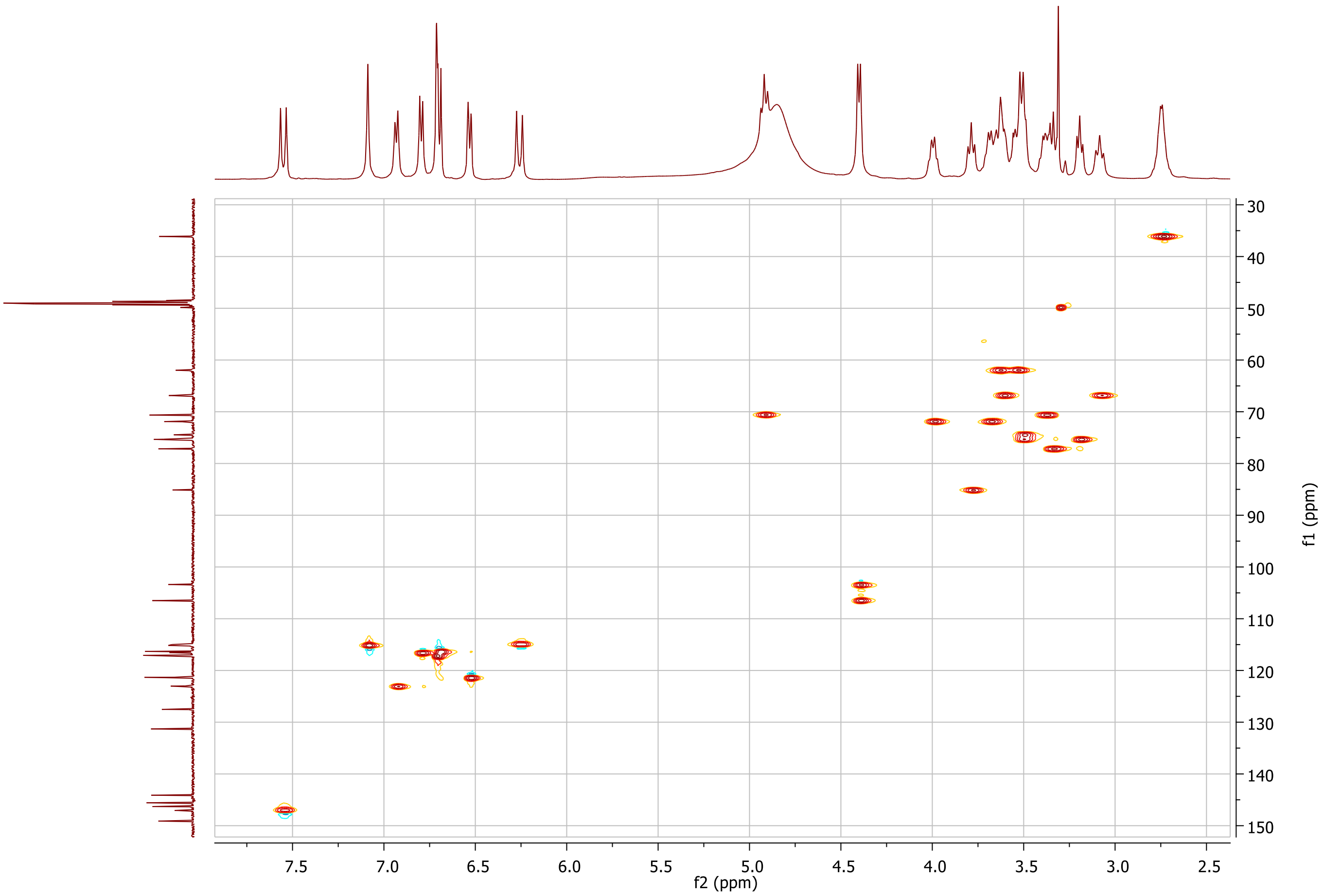

Figure S23 HSQC (500 MHz, CD3OD) spectrum of compound **5**

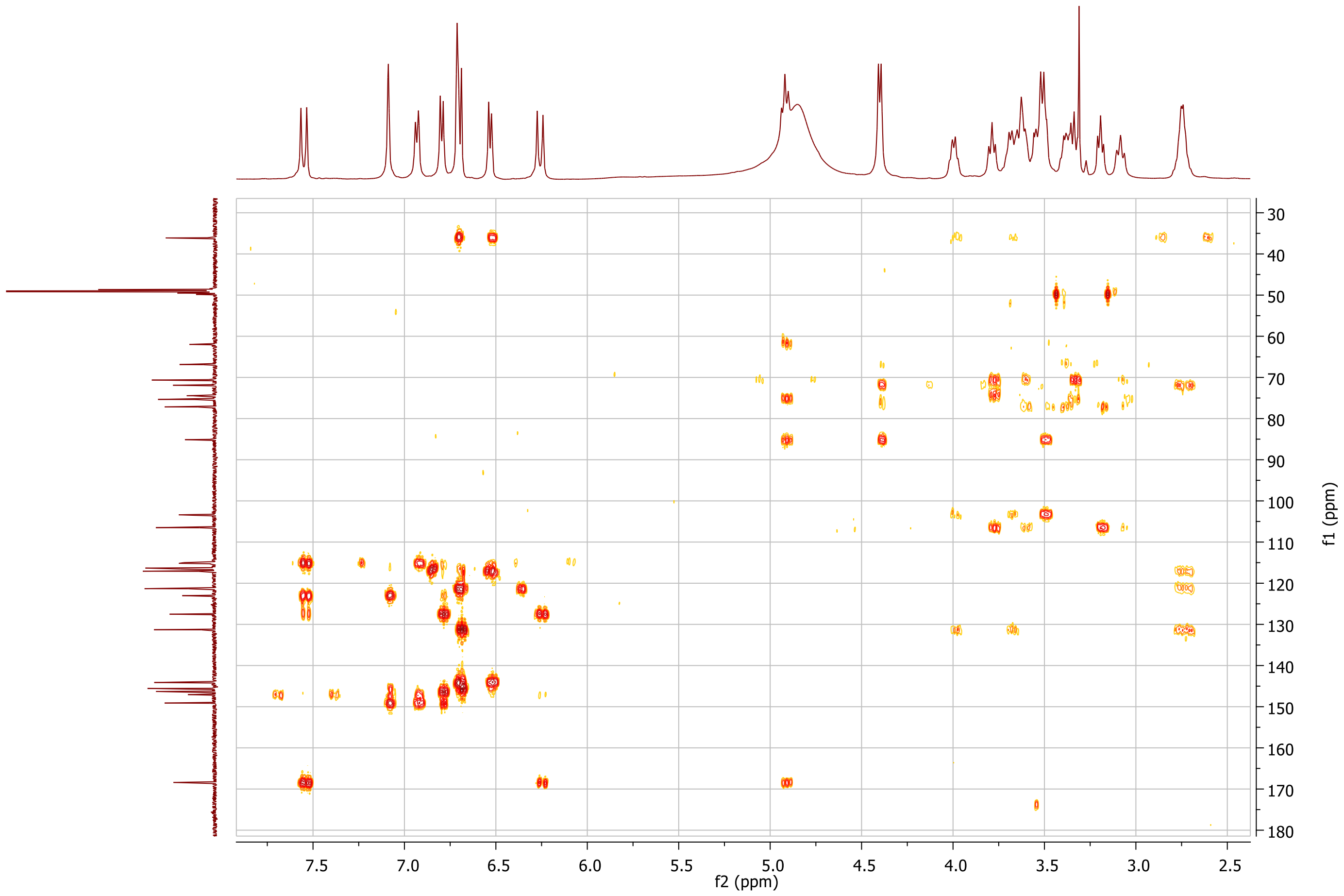

Figure S24 HMBC (500 MHz, CD3OD) spectrum of compound **5**
